# Supplementary material for: Changing the game of time resolved X-ray diffraction on the mechanochemistry playground by downsizing
Source: Nat Commun. 2021 Oct 21;12:6134. doi: 10.1038/s41467-021-26264-1 (PMC8531352; doi:10.1038/s41467-021-26264-1)
Supplement: Supplementary file 1 — Supplementary information. [file 41467_2021_26264_MOESM1_ESM.docx]

Supplementary Information

**Changing the game of time resolved X-ray diffraction on the mechanochemistry playground by downsizing**

Giulio I. Lampronti^*1,2^, Adam A. L. Michalchuk^*3^, Paolo P. Mazzeo^*4,5^, Ana M. Belenguer^1,3^, Jeremy K. M. Sanders^1^, Alessia Bacchi^4,5^, Franziska Emmerling^3^

1 Yusuf Hamied Department of Chemistry, University of Cambridge, Lensfield Road, Cambridge, CB2 1EW, UK

2 Department of Earth Sciences, University of Cambridge, Downing Street, Cambridge, CB2 3EQ, UK

3 Federal Institute for Materials Research and Testing (BAM), Richard-Willstätter-Str.11, D-12489 Berlin, Germany

4 Department of Chemistry, Life Sciences and Environmental Sustainability, University of Parma, Parco Area delle Scienze 17/A, 43124 Parma, Italy,

5 Biopharmanet-TEC, University of Parma, Parco Area delle Scienze 27/A, 43124 Parma, Italy

**SUPPLEMENTARY NOTE 1| METHODS**

## **Supplementary Note 1.1| Chemicals**

All starting materials used within this work were purchased from commercial suppliers and used without further purification.

- KI (ChemSolute) [7681-11-0] min. 99.5% purity.
- CsCl (Supelco, Merck) [7647-17-8] Suprapur, 99.995%
- ZnO (Acros Organic) [1314-13-2] was purchased at ACS reagent level of purity
- 2-Methyl-imidazole (Acros Organic) [693-98-1] 99% purity
- Ammonium nitrate (Merck, Emsure) [6484-52-2] was purchased as an ACS reagent
- Theophilline anhydrous (Acros Organics) [58-55-9] >99+%
- Benzamide (Aldrich) [55-21-0] 99%
- 1,8-Diazabicyclo[5.4.0]undec-7-ene (dbu) (Acros Organics) [6674-22-2] >97.5 %
- bis(4-chlorophenyl) disulfide (TCl) [1142-19-4] >98%; CofA lot FIA01= 99.7% (GC)
- bis(2-nitrophenyl) disulfide (TCl) [1155-00-6] >98%; CofA lot 25W4N= 99.9% (GC)

All solvents and acids used were obtained as follows:

- DMF [68-12-2] (CHEMSOLUTE, Germany) p.A. min. 99.9%
- Water was obtained from an in-house system in BAM. In the first step tap water is softened by a Grünbeck water softener. In the second step there softened water is passed through a reverse osmosis system from Nviro DTS
- MeCN [75-05-8 ] (CHEMSOLUTE, Germany), 99.8% HPLC grade
- Formic acid [64-18-6] Merck, Lichropur 98-100% for HPLC
- TFA [76-05-1] Merck, For LCMS

## **Supplementary Note 1.2| Materials**

All the ball bearings were sourced from Kugel-Winnie (Germany). The quality of the (hardened) stainless steel balls is EN 1.4043

## **Supplementary Note 1.3| Ex-situ measurements**

*HPLC analysis*

HPLC analysis to obtain the chemical composition of the disulfide reaction (Reaction IV) was performed using a modular Agilent 1200 Series HPLC system composed of a HPLC high pressure binary pump, autosampler with injector programming capabilities, Peltier type column oven with 6 µl heat exchanger and a Diode Array Detector with a semi-micro flow cell (1.6µl, 6mm pathlength) to reduce peak dispersion when using short columns as in this case. The flow-path was connected using 0.12 mm ID stainless steel tubing to minimize peak dispersion. This HPLC equipment was used in BAM.

For the disulfide analysis, the following HPLC conditions were used. A typical chromatogram is shown on **Supplementary Figure 1**.

- HPLC column: 1.8μm Agilent Zorbax SB C18, (4.6mm ID × 50 mm length)
- Solvent A: Water +0.1% Formic acid;
- Solvent B: Acetonitrile +0.1% Formic acid;
- Gradient of 0-2 minutes 75% - 85%B with re-equilibration time of 1 minute.
- Flowrate: 2 ml/min; Column temperature of 60^o^C;
- Injection volume of 1 μL

The signal was monitored at 260 nm (8 nm bandwidth) with reference at 550 nm (100 nm bandwidth). This wavelength and wavelength bandwidth was empirically selected as it gave the same peak area for (2NO_2_PhS)_2_ as for (4ClPhS)_2_ HPLC peaks, when an equimolar mixture of the homodimers had been accurately prepared. The peak area for (2NO_2_PhS)_2_, (4ClPhS)_2_ and 2NO_2_PhSSPh4Cl should be added together to give a total peak area, and the percent of the peak area of each peak will give the % molar. This is how the chemical composition in **Supplementary Table 1** has been calculated.

The disulfide sample was dissolved in MeCN + 0.2%TFA at a concentration of 1mg/ml (see **Supplementary Table 1**).

Note: The acid media from the addition of TFA in MeCN avoids the disulfides from exchanging and scrambling forming a statistical mixture of 1:1:2 of the disulfide homodimers ((2NO_2_PhS)_2_ and (4ClPhS)_2_) to the heterodimer 2NO_2_PhSSPh4Cl.^1^


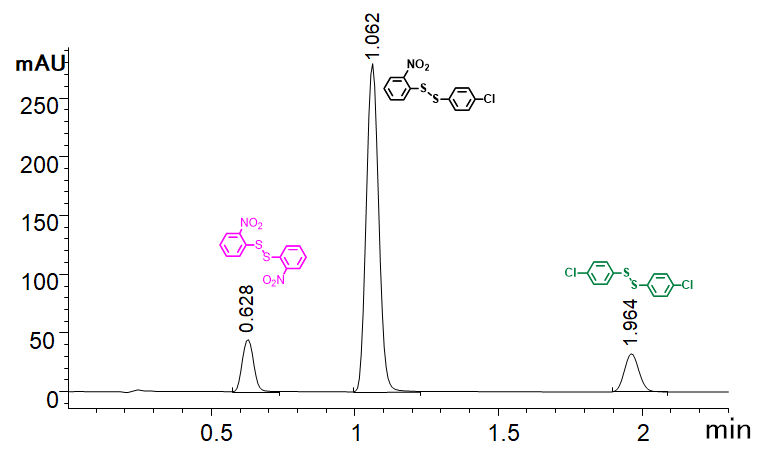


**Supplementary Figure 1**| Typical HPLC chromatogram of an enriched heterodimer 2NO_2_PhSSPh4Cl (made up of Form A + Form B). HPLC run contains the two homodimers, (2NO_2_PhS)_2_ and (4ClPhS)_2_.

## **Supplementary Note 1.4| In Situ Synchrotron X-ray Powder Diffraction**

Time resolved *in situ* (TRIS) X-ray powder diffraction (XRPD) data were collected at $\mu$Spot (BESSY-II, Helmholtz Zentrum Berlin). Data were collected at incident energies of 17 keV using a Si(111) monochromator. TRIS diffraction profiles were collected using accumulation times of 5 s per frame. Scattering was collected on an Eiger 9M 2D detector. A beam size of 150 $\mu$m diameter was used. Sample-to-detector distance was typically set at *ca.* 250 mm.

We note that the Eiger 9M detector was covered by a 50 $\mu$m polyimide (Kapton) shield to protect against accidental damage from the milling equipment. Although this polyimide shield contributes to scattering, **Supplementary Figure 2**, its intensity falls well below that of the milling jar and powdered sample. It is therefore not observed during TRIS XRPD monitoring.


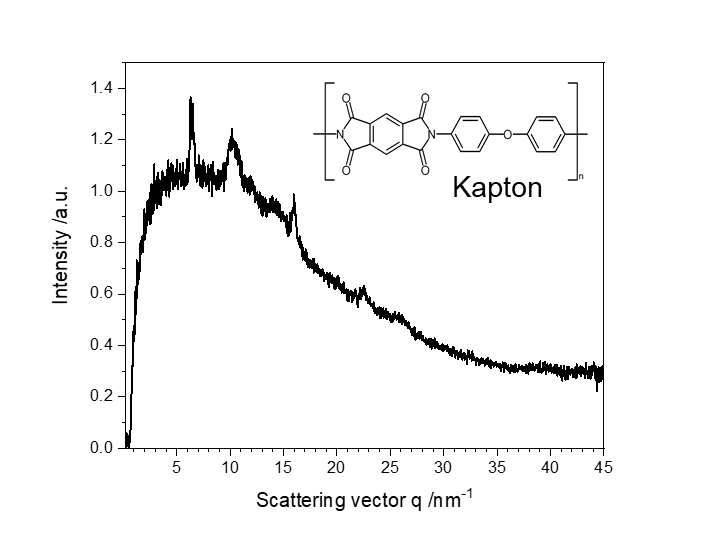


Intensity (arb. units)

**Supplementary Figure 2|** Integrated X-ray scattering profile for the polyimide (Kapton) shield present in all TRIS XRPD experiments performed in this work.

## **Supplementary Note 1.5| Equipment**

*Ball mill grinder*

The ball mill grinding experiments at BESSY II were all performed using a Fritsch P23 vertical movement Shaker Mill, **Supplementary Figure 3**. This mill has a fixed amplitude of 9 mm and adjustable frequency from 15 Hz to 50 Hz with an adjustable timer. An adaptor was made in house (BAM) to ensure that the milling jar were kept in position during grinding. Additionally, a horizontal movement Retsch MM400 was used to distribute dbu into bis-4-chlorophenyldisulfide for Reaction IV as explained in **Supplementary Note 4.4**.^1^


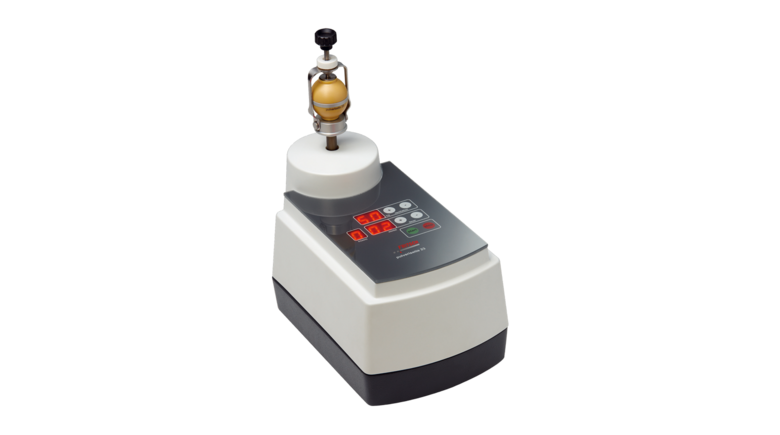


**Supplementary Figure 3**| Fritsch Pulverisette 23 (P23) in its standard configuration with commercial jar included.

# **SUPPLEMENTARY NOTE 2| MILLING JAR DESIGN**

All experiments were conducted using a custom design of milling jars prepared at the mechanical workshop at BAM. Two different milling jar set-ups were used (See 3 part 2.3mL “small” milling jar in **Supplementary Figure 5** and 2-part 1.8 mL “tiny” milling jar **in Supplementary Figure 7**.)

Additionally, a 46 mL custom built (Department of Chemistry, University of Cambridge) stainless steel snap close grinding jar was used to mix dbu with bis-4-chlorophenyldisulfide for Reaction IV as explained in **Supplementary Note 4.4**.

## **Supplementary Note 2.1| X-ray transmission of Perspex (PMMA)**

Perspex has been traditionally selected for TRIS mechanochemical investigations owing to its low absorption coefficient for X-rays. Lower energy X-rays are less penetrating through organic phases and hence careful consideration of jar thickness at these energies was critical. Simulated absorption coefficients, **Supplementary Figure 4**, suggest that 0.5 mm Perspex absorbs only *ca.* 5% of incident photons at 17 keV, increasing to *ca.* 10% by 1 mm. At 17 keV, we therefore expect our milling jars (2 x 0.75 mm walls and 2 x 0.5 mm walls) to absorb *ca.*17% and 10% of the incident photons for the small and tiny jars, respectively.


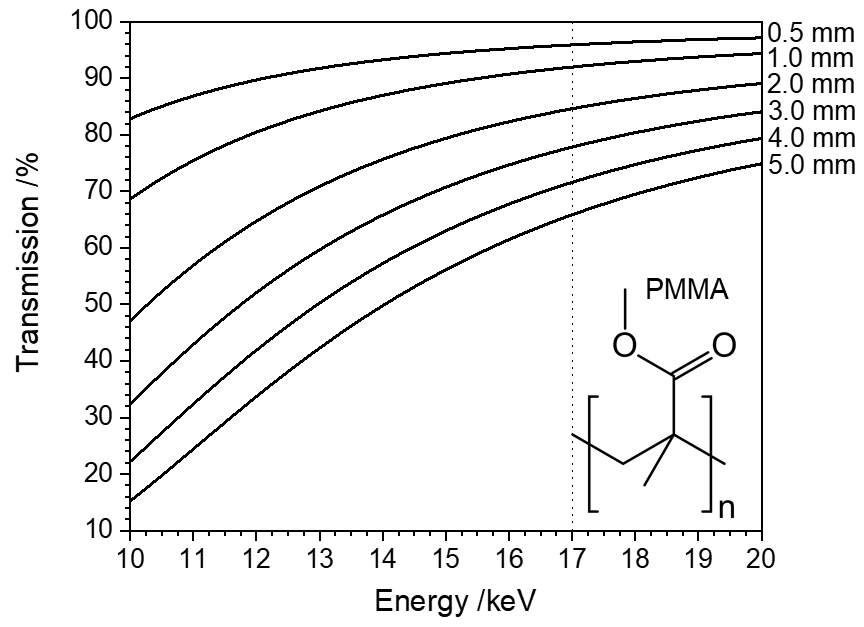


**Supplementary Figure 4** | Simulation of X-ray transmission through various thicknesses of Perspex (PMMA, C_5_H_8_O_2_, density 1.19 g.cm^-3^).

## **Supplementary Note 2.2| 2.3 mL 3-part grinding Jar - Small jar**

The 2.3 mL jar is comprised of three pieces, two stainless steel or polyvinyl chloride (PVC) end pieces and a transparent Perspex middle segment of 0.75 mm thickness as shown in **Supplementary Figure 5**. The end pieces were made to have hemispherical internal geometry to allow access of the milling ball to all areas of the internal volume. The overall dimensions of the milling jar are shown in **Supplementary Figure 5b**). The walls of the Perspex piece were 0.75 mm in thickness, and its X-ray diffraction profile is shown in **Supplementary Figure 6**. This jar was used for reactions I-III.


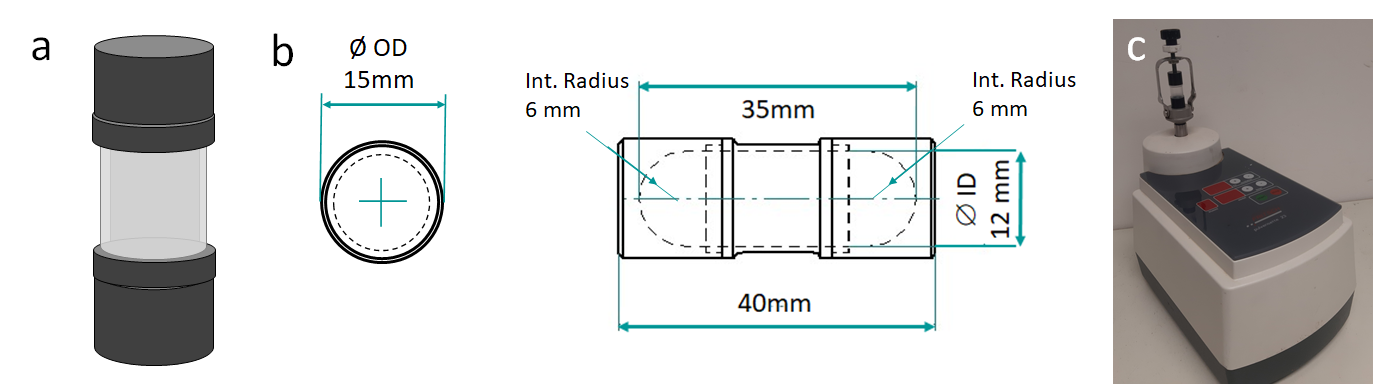


**Supplementary Figure 5**| Custom built 2.3 mL grinding jar; a) the central Perspex transparent cylindrical part is snap-closed to the top and bottom hemispheric caps made either of stainless steel or polyvinyl chloride; b) mechanical drawing of this jar; c) photograph of the jar installed into the Fritsch P23 vertical vibratory mill.


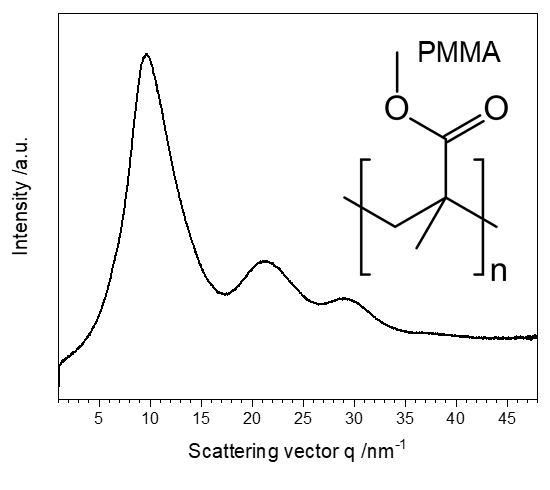


Intensity (arb. units)

**Supplementary Figure 6**| Integrated diffraction profile for the empty 2.3 mL small Perspex (PMMA) jar

## **Supplementary Note 2.3| 1.8 mL 2-part grinding Jar - Tiny jar**

The 1.8 mL jar was composed of two pieces: a long Perspex shaft with a PVC lid. The ends were shaped into hemispheres to allow access of the milling ball to the whole internal volume of the jar. The overall dimensions of the milling jar are shown in **Supplementary Figure 7b**. The wall thickness of the Perspex is 0.5 mm, and the corresponding X-ray diffraction profile is shown in **Supplementary Figure 8**. This jar was used for reaction IV.


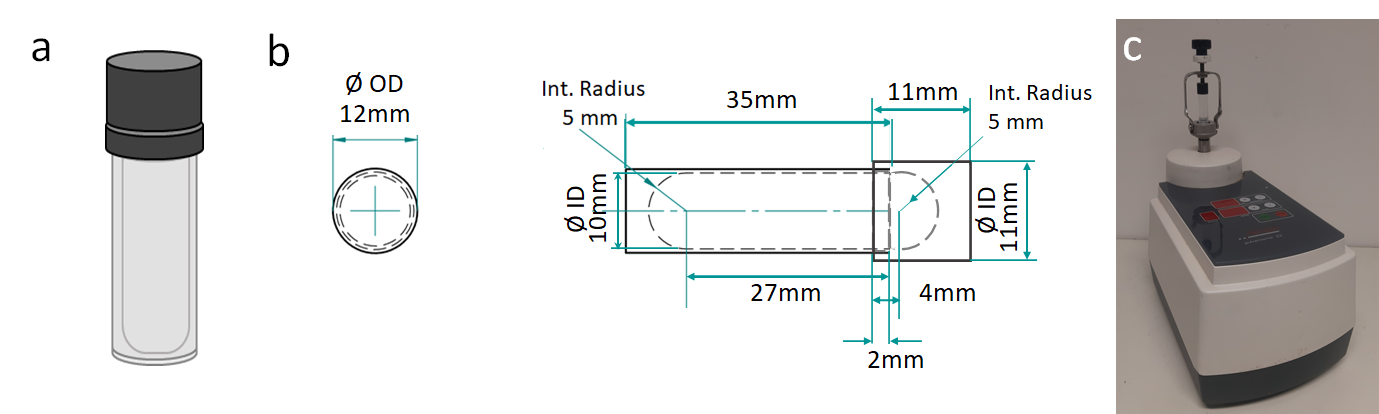


**Supplementary Figure 7**| Custom built 1.8 mL grinding jar; a) the central Perspex transparent cylindrical part is snap-closed to the top hemispheric caps made of polyvinyl chloride; b) mechanical drawing of this jar; c) photograph of the jar installed into the Fritsch P23 vertical vibratory mill.


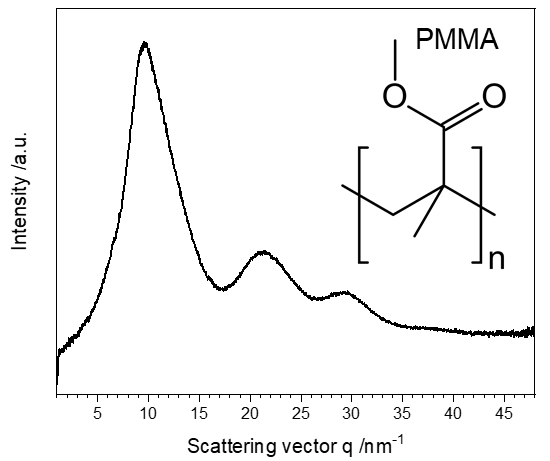


Intensity (arb. units)

**Supplementary Figure 8**| Integrated diffraction profile for the empty 1.8 mL tiny Perspex (PMMA) jar

# **SUPPLEMENTARY NOTE 3| DATA ACQUISITION STRATEGY**

## **Supplementary Note 3.1| Jar alignment**

Prior to each TRIS experiment, the sample jar was loaded into the mill and aligned. A photodiode was placed behind the milling jar and the intensity of the impinging X-ray beam was measured. The milling apparatus was moved in steps of 100 $\mu$m through the beam and the absorption of radiation by the jar was measured on the photodiode, **Supplementary Figure 9**. With a beam size of 150 $\mu$m, this step size ensured overlap between adjacent sampling points for best coverage of the jar geometry. The jar wall is clearly visible on both sides of the jar, where the material density increases and hence the diode reading decreases.


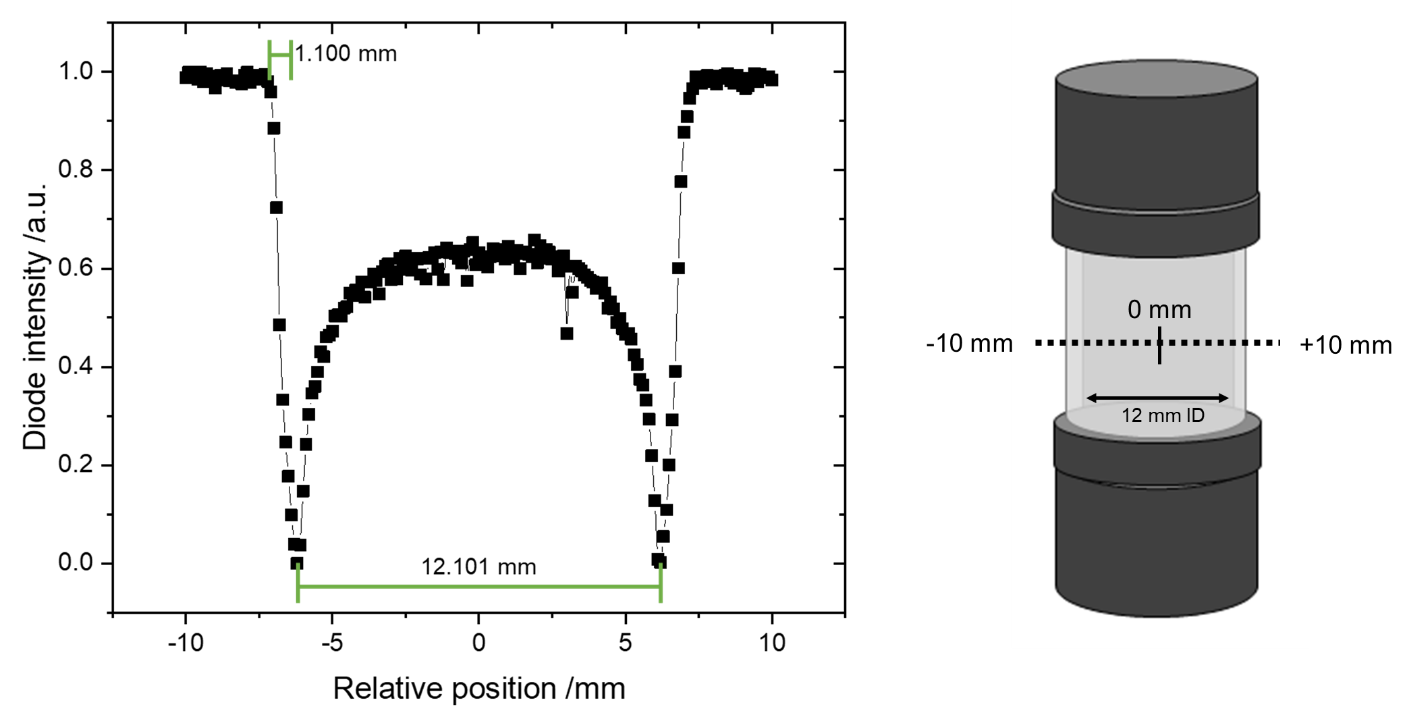


Diode intensity (arb. units)

**Supplementary Figure 9**| Photodiode alignment of the small custom built Perspex milling jar in the X-ray beam. (Left) The milling jar is mapped by the absorption of X-ray intensity as it passes through the milling jar and is scanned along a (right) horizontal path across the jar diameter. Note that the milling jar walls are clearly visible and are in excellent agreement with manufactured dimensions (see Supplementary Figure 5)

To identify the desired position, a powder of NIST Si 640d standard was placed into the milling jar with a 5 mm stainless steel milling ball and shaken at 50 Hz. 2D X-ray diffraction profiles were collected at 100 $\mu$m steps through the milling jar, **Supplementary Figure 10**. Note that the steps were selected to reproduce exactly the map across the empty vessel (**Supplementary Figure 9**). It is clearly visible that only at the extreme edges of the vessel is a quasi-single Bragg reflection of the Si standard observed. Splitting of the reflections increases quickly as the beam is moved away from the extreme edge, morphing into the complex diffraction profile (see **Figure 2** in main manuscript) that results from multiple scattering centres. For collection of TRIS XRPD data in this work, alignments were selected at the extreme edges to best reproduce singlet diffraction profiles (see **Figure 1** in the main manuscript).


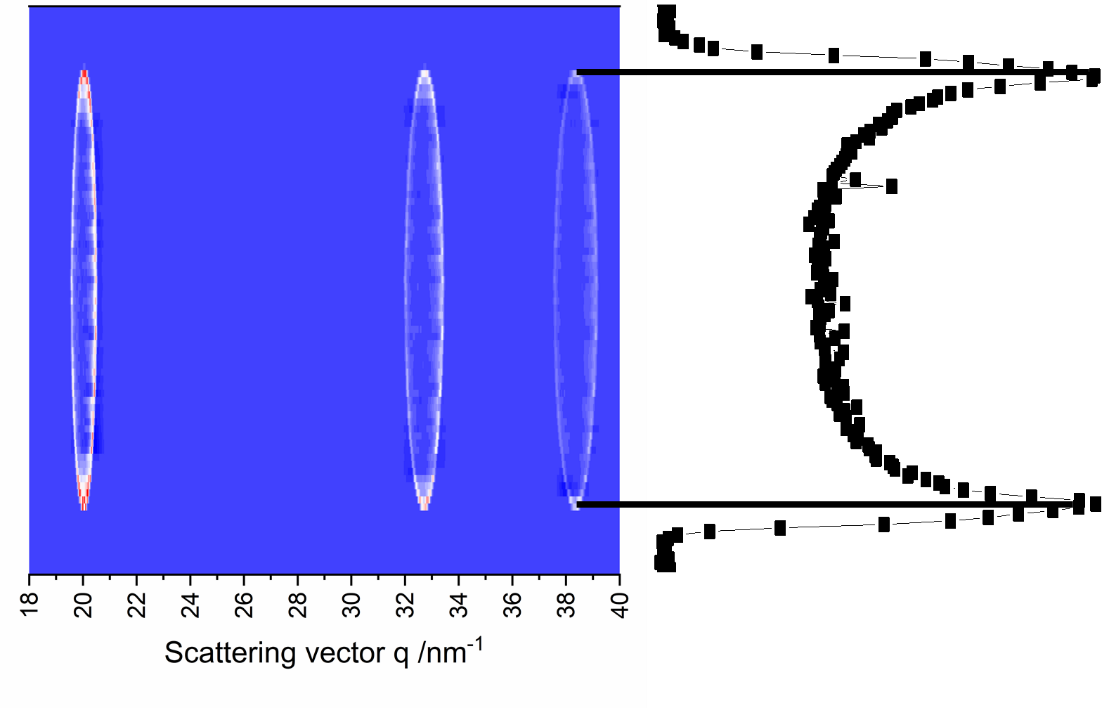


**Supplementary Figure 10|** Effects of alignment on the diffraction profile of a NIST Si640d standard. Diffraction profiles were measured at 100 $\mu$m steps across the horizontal axis of the small custom built milling jar (see Supplementary Figure 5).

# **SUPPLEMENTARY NOTE 4| Time Resolved PXRD applications**

## **Supplementary Note 4.1| Reaction I: inorganic metathesis reaction by neat grinding**


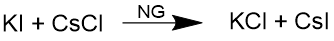


**Supplementary Figure 11** | Chemical equation for reaction I via neat grinding

To avoid mixing the starting materials KI and CsCl, **Supplementary Figure 11**, before starting the grinding experiment, each starting material was weighed separately and mixed just before starting the TRIS monitored ball mill neat grinding experiment at the synchrotron.

To obtain a loading of 70 mg, 1 equivalent of CsCl (0.21 mmol, 35.36 mg) was weighed on a 5-figure balance directly inside the bottom stainless-steel cap of the 2.3 mL little jar. The transparent middle part was snap tight on top, and the system was sealed with a PVC snap cap on top of the transparent segment. 1 equivalent of KI (0.21 mmol, 34.96 mg) was weighed on a 5-figure balance directly inside the top stainless-steel cap of the 2.3 mL little jar. To ensure safe transport and that the KI was kept dry, a middle Perspex segment was snap closed to the top cap and a PVC cap was snap closed to the open part of the transparent plastic segment. The 8 mm stainless steel ball was stored inside an Eppendorf plastic vial for transport.

Just before starting the ball mill grinding experiment, the PVC cap was removed from the bottom stainless-steel cap snapped to the central Perspex segment containing CsCl. The 8 mm ball was added and the KI powder contained in the top stainless-steel cap was quantitatively transferred to the grinding jar and sealed immediately with the top stainless-steel cap. Following addition of all reaction components and the milling ball, the jar was closed and installed on the P23. Jar alignment was carried out (see **Supplementary Note 3**) and the ball milling reaction started at 50 Hz. The reaction was continued until no further changes in the XRPD profiles were visible.

## **Supplementary Note 4.2| Reaction II: Synthesis of ZIF-8 by LAG with DMF**

**Supplementary Figure 12**| chemical equation for reaction II via liquid assisted grinding protocol

To avoid mixing the starting materials, **Supplementary Figure 12**, ZnO and both 2-methyl-imidazole and ammonium nitrate were weighed separately and only mixed with the addition of DMF just before starting the *in situ* monitored ball mill LAG grinding experiment at the synchrotron.

To obtain a loading of 70 mg, 2 equivalents of 2-methyl-Iimidazole (2x0.277 mmol, 45.48 mg) was weighed on a 5-figure balance directly inside the bottom stainless-steel cap of the 2.3 mL little jar. 0.1 equivalent of ammonium nitrate (0.0277 mmol, 2.22 mg) was weighted on a small weighing boat prepared from grease proof weighing paper and transferred quantitatively to the bottom cap already containing 2-methyl-imidazole. The transparent middle part was snap tight on top, and the system was sealed with a PVC snap cap. 1 equivalent of ZnO (0.277 mmol, 22.54 mg) was weighed on a 5-figure balance directly inside the top stainless-steel cap of the 2.3 mL little jar. To ensure safe transport and that the ZnO was kept dry, a middle Perspex segment was snap closed to the top cap and a PVC cap was snap closed to the open part of the transparent plastic segment. The 8 mm stainless steel ball was stored inside an Eppendorf plastic vial for transport.

Just before starting the grinding, the top PVC cap was removed from the bottom stainless-steel cap snapped to the central Perspex segment containing 2-methyl-imidazole and NH_4_NO_3_., The 8 mm ball was added to the bottom cap. ZnO powder contained in the top cap was quantitatively transferred to the grinding jar already containing 2-methyl-imidazole and NH_4_NO_3_. 17 μL DMF was added on top of the powder (using 20μl pipette) and the tip rested on the ball bearing. The middle transparent segment was sealed with the top stainless cap immediately. Following addition of all reaction components and the milling ball, the jar was closed and installed on the P23. Jar alignment was carried out (see **Supplementary Note 3**) and the ball milling reaction was started at 50 Hz. The reaction was continued until no further changes in the XRPD profiles were visible.

## **Supplementary Note 4.3| Reaction III: Synthesis of Theophylline:Benzamide cocrystals**


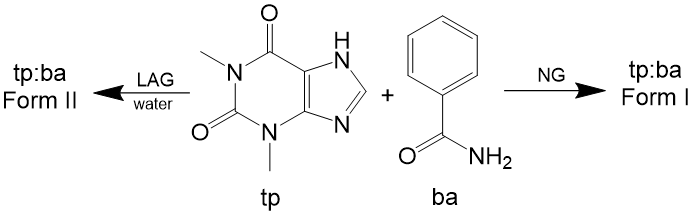


**Supplementary Figure 13**| chemical equation for reaction III. tp:ba Form I is obtained via neat grinding while tp:ba Form II is obtained via liquid assisted grinding.

To avoid mixing the starting materials, **Supplementary Figure 13**, anhydrous Theophylline anhydrous and Benzamide were weighed separately for NG to obtain Form I cocrystal (**Supplementary Note 4.3.1**) and for LAG with water to obtain Form II cocrystal (**Supplementary Note 4.3.2**). The starting materials were mixed just before starting the in-situ monitored ball mill grinding at the synchrotron. Nothing extra was added for NG, while 9μL water was added for LAG just before starting the experiment. Following addition of all reaction components and the milling ball, the jar was closed and installed on the P23. Jar alignment was carried out (see **Supplementary Note 3**) and the ball milling reaction started at 50 Hz. The reaction was continued until no further changes in the XRPD profiles were visible.

**Supplementary Note 4.3.1| Synthesis of 1:1 Theophylline: Benzamide Form I by NG**

To obtain a loading of 60 mg, 1 equivalent of Theophylline anhydrous (0.20 mmol, 36.03 mg) was weighed on a 5-figure balance directly inside the bottom stainless-steel cap of the little 2.3 mL jar. The transparent middle part was snap tight on top, and the system was sealed with a PVC snap cap on top of the transparent segment to ensure that Theophylline anhydrous did not pick up humidity. 1 equivalent of Benzamide (0.20 mmol, 24.23 mg) was weighed on a 5-figure balance directly inside the top stainless-steel cap of the little jar. To ensure safe transport and that the Benzamide was kept dry, a middle Perspex segment was snap closed to the top stainless-steel cap and a PVC cap was snap closed to the open part of the transparent plastic segment. The 7 mm stainless steel ball was stored inside an Eppendorf plastic vial for transport.

Just before starting the grinding, the top PVC cap was removed from the bottom -steel cap snapped to the central Perspex segment containing benzamide. The 8 mm ball was added to the powder on the bottom stainless-steel cap. The theophylline anhydrous powder contained in the top stainless-steel cap was quantitatively transferred to the grinding jar containing already benzamide. 9 μL water was added on top of the powder (using 10μL pipette) and the tip rested on the ball bearing. The middle transparent segment was sealed with the top stainless cap immediately.

**Supplementary Note 4.3.2| Synthesis of 1:1 Theophylline: Benzamide Form II by LAG with water**

To obtain a loading of 60 mg, 1 equivalent of theophylline anhydrous (0.20 mmol, 36.02 mg) was weighed on a 5-figure balance directly inside the bottom stainless-steel cap of the little 2.3 mL jar. The transparent middle part was snap tight on top, and the system was sealed with a PVC snap cap on top of the transparent segment to ensure that anhydrous theophylline did not pick-up humidity. 1 equivalent of Benzamide (0.20 mmol, 24.23 mg) was weighed on a 5-figure balance directly inside the top stainless-steel cap of the little jar. To ensure safe transport and that the Benzamide was kept dry, a middle Perspex segment was snap closed to the top stainless-steel cap and a PVC cap was snap closed to the open part of the transparent plastic segment. The 7 mm stainless steel ball was stored inside an Eppendorf plastic vial for transport.

Just before starting the grinding, the top PVC cap of the bottom plus central Perspex segment containing benzamide was removed, the 8 mm ball was added to the bottom cap and the theophylline anhydrous powder contained in the top stainless-steel cap was quantitatively transferred to the grinding jar. The middle transparent segment was sealed with the top stainless-steel cap immediately.

## **Supplementary Note 4.4| Reaction IV: Synthesis of Disulfide exchange reaction by neat grinding**


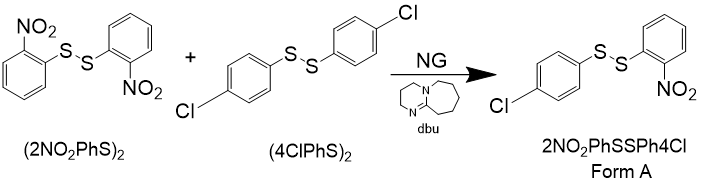


**Supplementary Figure 14**| Chemical equation for reaction IV via neat grinding

The disulfide reaction was prepared at a 10 mg scale by ball mill neat grinding using the 1.8mL tiny 2-part grinding jar. This is a base catalysed, disulfide exchange reaction using equimolar amounts of bis-2-nitrophenyldisulfide (2NO_2_PhS)_2_ and bis-4-chlorophenyldisulfide (4-ClPhS)_2_, in the presence of a small amount (1-2%M) of base catalyst 1,8-Diazabicyclo[5.4.0]undec-7-ene (**dbu**) to produce the compound 4-chlorophenyl-2-nitrophenyl-disulfide (4-ClPhSSPhNO_2_) in quantitative yield, **Supplementary Figure 14.**^1^ Under neat grinding conditions polymorph Form A is obtained.

For a 10 mg scale, we would have required 0.016 mmol of each of the homodimers: 4.93mg of (2NO_2_PhS)_2_ and 4.60mg of (4ClPhS)_2_ with 2%M of dbu (0.00064 mmol, 0.1μl). Dbu is a very viscous liquid (d=1.018 g/ml). Even with an accurate 10μl pipette, 0.1μl of dbu is much too difficult to dispense. As dbu is used catalytically, even 1%M would be equally effective.

Therefore, we opted to dispense 4%M dbu directly to (4ClPhS)_2_**.** We prepared a mixture of 2 g (6.96 mmol) of (4ClPhS)_2_ with 42μl dbu (0.28 mmol) into a 46 ml large-scale stainless-steel snap closure grinding jars with two ½ inch ID hardened stainless steel ball bearings (each ball 8.22g), and performed ball mill neat grinding on a MM400 for 15 min at 25 Hz to ensure that dbu was homogenously mixed in (4ClPhS)_2._ This sample was analysed by HPLC against the commercially available material taken as an external reference standard. The content of (4ClPhS)_2_ in this sample containing dbu was determined to be 97.9% as compared to the commercially available product assumed to be 100%.

4.86 mg (expected 4.94mg, 0.016mmol) of (2NO_2_PhS)_2_, 99.9% pure as per manufacturer’s CofA was weighed into the tiny 1.8 mL grinding jar using a 5-figure balance. For (4ClPhS)_2_, a slightly larger amount would be required to make both homodimers equimolar. Taking into account that (4ClPhS)_2_, is 99.7% pure as per manufacturer’s CofA and that the content of (4ClPhS)_2_ is 97.9% in the mixture with dbu, 4.71mg corresponding to 0.0164 mmol should be weighed. Therefore 4.71mg of the mixture (4ClPhS)_2_ plus dbu was weighed in a grease-proof paper with a 5-figure balance and quantitatively transferred to a 1.8 mL tiny Perspex jar already containing (2NO_2_PhS)_2_. One 6 mm stainless steel ball was added to the jar. Following addition of all reaction components and the milling ball, the jar was closed and installed on the P23. Jar alignment was carried out (see **Supplementary Note 3**) and the ball milling reaction started at 50 Hz. The reaction was continued until no further changes in the XRPD profiles were visible.

# **SUPPLEMENTARY NOTE 5| DIFFRACTION DATA ANALYSIS**

## **Supplementary Note 5.1| Rietveld refinements, quantitative analysis, and microstructural analysis**

*General Refinement details*

The evolution of the phases through the reaction was followed by Quantitative Phase Analysis (QPA) performed by Rietveld Refinement. Our TRIS setup induces aberration in terms of the peak profile and peak positions (*i.e.* the actual 2θ peak position with respect to the expected Bragg reflection; see e.g. **Supplementary Figure 10**). We discuss here the analytical details for the refinement strategy used throughout this work.

*Sample displacement aberration correction*

A cylindrical jar oscillates vertically and perpendicularly with respect to the beam path. The beam crosses the jar and the final XRPD pattern thus results as a convolution of the scattering that originates from the sample attached to the jar walls (namely $\vec{s_{1}}$ and $\vec{s_{3}}$, see **Figure 1** in the main manuscript) and from the sample distributed randomly within the jar $\vec{s_{2}}$. In Debye-Scherrer (transmission) geometry, the offset of the sample with respect to the ideal centre of the goniometer causes a nonlinear shift in the 2θ positions calculated as **Supplementary Equation 1**^2^

| $\Delta2\theta_{sd}=\arcsin\left( \frac{ⅆ_{L}}{R_{DS}}\sin2\theta\right)-\arcsin\left( \frac{ⅆ_{V}}{R_{DS}}\cos2\theta\right)$ | Supplementary Equation 1 |
| --- | --- |

where $ⅆ_{L}$ and $ⅆ_{V}$ denote, respectively, the displacement of the sample in the direction of the beam and perpendicular to it and $R_{DS}$ is the sample-to-detector distance.

In the Rietveld Refinement, each scattering vector (schematized in **Figure 1** in the main text) is defined as a unique phase with the lattice parameters constrained to be identical among the three of them. A longitudinal sample displacement correction $ⅆ_{L}$ is individually applied for each scattering vector. Assuming that the powder distributes homogeneously along the vertical direction, the vertical sample displacements do not influence further the angular offset. Correspondingly, the final correction is applied according to **Supplementary Equation 2** (see macro Peak_split_simple in **Supplementary Note 8**)

| $\Delta2\theta_{hkl}=\arcsin\left( \frac{ⅆ_{L}}{R_{DS}}{\sin2\theta}_{hkl} \right)$ | Supplementary Equation 2 |
| --- | --- |

*Peak shape definition*

The dependence of the profile full-width-at-half-max (FWHM) on 2θ was described with a modified Thompson-Cox-Hasting^3^ pseudo-Voigt TCHZ function where U, V, W (Gaussian) and X (Lorenzian) are the parameters that have been refined in the current case.^4^

| $TCHZ=\eta\Gamma_{L}+\left( 1-\eta\right)\Gamma_{G}$ | Supplementary Equation 3 |
| --- | --- |
|  |  |
| $\Gamma_{G}=\sqrt{\left( U{tan}^{2}\theta+Vtan\theta+W+Z/{cos}^{2}\theta\right)}$ | Supplementary Equation 4 |
|  |  |
| $\Gamma_{L}=\frac{X}{cos\theta}+Ytan\theta$ | Supplementary Equation 5 |
|  |  |
| $\eta=1.336603 q-0.47719 q^{2}+0.1116 q^{3}$ | Supplementary Equation 6 |
|  |  |
| $q=\Gamma_{L}/ \Gamma$ | Supplementary Equation 7 |
|  |  |
| $\Gamma=\sqrt{\Gamma_{G}^{5}+A{\Gamma_{G}^{4}\Gamma}_{L}+{B\Gamma}_{G}^{3}\Gamma_{L}^{2}+{C\Gamma}_{G}^{2}\Gamma_{L}^{3}+{D\Gamma}_{G}\Gamma_{L}^{4}+\Gamma_{L}^{5}}=FWHM$ | Supplementary Equation 8 |
|  |  |
| $A=2.69269; B=2.42843;C=4.47163;D=0.07842$ | Supplementary Equation 9 |

Where $\eta$ is the Pseudo-Voigt mixing parameter, and $\Gamma_{G}$ and $\Gamma_{L}$are the Gaussian and Lorentzian full width half maxima, respectively. Additionally, to model the evident peak asymmetry, the TCHZ was further split to differentiate the contribution at the left-hand side and at the right-hand site to the overall peak profile for the inner and outer scattering vectors $\vec{s_{1}}$ and $\vec{s_{3}}$.^5^ The final TCHZ_split_ function is applied to describe the overall dependence of the FWHM according to **Supplementary Equation 10** (see macro TCHZ_Split_Peak_Type in **Supplementary Note 8**),

| ${TCHZ}_{split}=\left( \eta\Gamma_{L}+\left( 1-\eta\right)\Gamma_{G} \right)_{Left}+\left( \eta\Gamma_{L}+\left( 1-\eta\right)\Gamma_{G} \right)_{Right}$ | Supplementary Equation 10 |
| --- | --- |

A further Gaussian function was convoluted to empirically describe the dependence of the peak FWHM on the actual size of the jar at the position of the beam. In other words, the FWHM of $\vec{s_{2}}$ is influenced by the length of the beam path through the jar (see e.g. **Figure 1** in the main manuscript).

NIST Si640d^6^ was used as a standard for the careful characterization of the Instrumental Resolution Function (IRF) under the same experimental conditions used for TRIS monitoring.

*Quantitative Phase Analyses and microstructural evolution analysis details*

Rietveld QPA of experimental diffraction patterns was performed using TOPAS-Academic V6.^7^ The structural models were retrieved from either the CSD or the ICSD database. XRPD datasets were fit sequentially, with a convergence criterion of 0.0005 and a maximum number of iterations of 1000. An experimental, fixed background profile measured on an empty section of the jar before milling, together with a seventh-order Chebychev polynomial. As described for the Si standard, the crystal structure model of each compound was introduced twice (“top phase” and “bottom phase” from here on) with two distinct TCHZ peak shape functions (with fixed parameters as refined on the Si standard) and independent scale parameters. The composition of the top phase assemblage and that of the bottom phase assemblage were kept equal by setting the scale factor of each bottom phase to be equal to that of the corresponding top phase with a multiplication factor common to all bottom phases. Microstructural investigations were performed assuming that the sample contribution to peak broadening was related to size only. A Lorentzian function was convoluted for each phase, with a single isotropic Crystal Size (CS) parameter related to $\Gamma_{L}$ as in the Scherrer equation (**Supplementary Equations 11 and 12**),^8^

| $L(nm)=\frac{K_{s}\lambda}{\left( cos\theta\right)*10*\tau}$ | Supplementary Equation 11 |
| --- | --- |
|  |  |
| $\Gamma_{L}=\frac{57.32*\lambda}{cos\theta*CS}$ | Supplementary Equation 12 |

in which, *L* is the mean size of the ordered (crystalline) domains, *K_s_* is a shape factor constant in the range (typically 0.9), *λ* is the X-ray wavelength, *τ* is the peak width in radians at FWHM. The top and bottom phases of the same compound were constrained to have the same CS parameter. We here remind that the estimated standard deviation (ESD) from the Rietveld calculation has no bearing on the precision or accuracy, but is merely related to the mathematical fit of the model.^9^ In other words, absolute numbers have a degree of uncertainty that cannot really be measured. On the other hand, so long as the same approach is used for all scans within a dataset, trends are reliable. For what concerns the accuracy of the size determination, it is known that for a typical laboratory X-ray diffraction instrument the Scherrer analysis provides sensitivity to crystallite size in the 1–100 nm range, the upper limit being set by the instrumental broadening.^8^ This also means that the smaller the crystal size, the less the Scherrer size value is affected by how the instrumental broadening is defined. For example, modeling the instrumental contribution for the analyzed datasets with a single conventional TCHZ function, instead of two split TCHZ functions, has a more significant effect on the estimated CS for larger CSs. As an example, we can discuss the case of the cocrystallisation of theophylline and benzamide. For Form II of the 1:1 co-crystal of theophylline and benzamide (Reaction IIIb, scan 060), the estimated CS is reduced from 262 (± 20) nm using two split TCHZ to model the instrumental contribution to peak broadening, to 123 (± 5) nm using a single TCHZ. Similarly, when considering the co-former theophylline (Reaction IIIa, scan 015), the estimated CS is reduced from 191 (± 64) nm to 142 (± 18) nm when reducing from two to one TCHZ fitting function. In the case of Form I of the 1:1 co-crystal of theophylline and benzamide (Reaction IIIa, scan 300), using only one TCHZ function instead reduces the crystal size from 92 (± 4) nm to 69 (± 1) nm. However, for the co-former benzamide (Reaction IIIa, scan 015), the change in estimated CS is relatively small on reducing from two split TCHZ to one TCHZ fitting function, changing from 35 (± 6) nm to 37 (± 6) nm. Hence, benzamide CS is virtually unaffected by how the instrumental broadening is described. To summarize, the smaller the crystal size, the more reliable the number. It is also important to note that the peak shape tends to be dominated by the larger crystallites rather than the smaller ones, so the calculated size tends to be overestimated.^8^

While the visual inspection of a Rietveld plot is the most reliable way to determine the quality of a fit, this is not practical for large datasets, such as those presented here. A global check of a sequential refinement can be efficiently performed by comparing a number of “goodness of fit” indices. One is the weighted profile R-factor (*R_wp_*),

$R_{wp}^{2}= \frac{\sum_{i} w_{i}\left( y_{c,i}-y_{o,i} \right)^{2}}{\sum_{i} w_{i}\left( y_{o,i} \right)^{2}}$ Supplementary Equation 13

Where *y_c_* and *y_o_* represent the calculated and observed intensity respectively for each point *i*. and the weight *w_i_* is equal to $1/\sigma^{2}\left[ y_{o,i} \right]$. The second index is “chi squared”:

$\chi^{2}= \left( \frac{R_{wp}}{R_{exp}} \right)^{2}$ Supplementary Equation 14

where (*R_exp_*), the “expected *R* factor”, is:

$R_{exp}^{2}= \frac{N}{\sum_{i} w_{i}\left( y_{o,i} \right)^{2}}$ Supplementary Equation 15

with *N* as the number of data points.

## **Supplementary Note 5.2| Reaction I – inorganic metathesis reaction**

Lattice parameters, scaling factors and CS parameters were refined individually for all scans. The scale factors of KCl and CsI were constrained to keep their stoichiometric ratio as 1:1. **Supplementary Figure 15** shows plots of the *R_wp_* and *χ^2^* for all the scans as obtained from the sequential Rietveld refinement. **Supplementary Figure 16** shows representative Rietveld plots of three scans at (a) the start of the experiment, (b) the midpoint of the experiment, and (c) the end of the experiment.


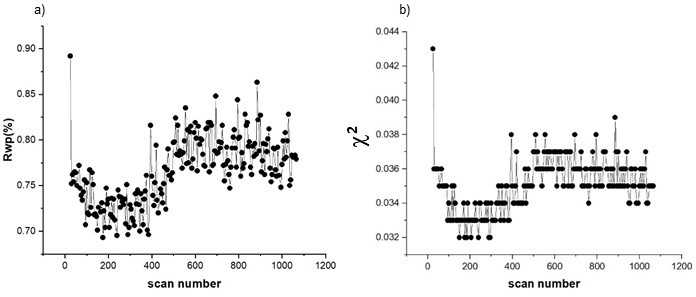


**Supplementary Figure 15** | Fit statistics for Rietveld refinement of the inorganic metathesis reaction (Reaction I), showing R_wp_ (a; Supplementary Equation 13) and χ^2^ (b; Supplementary Equation 14) for all the scans as obtained from the sequential Rietveld refinement.


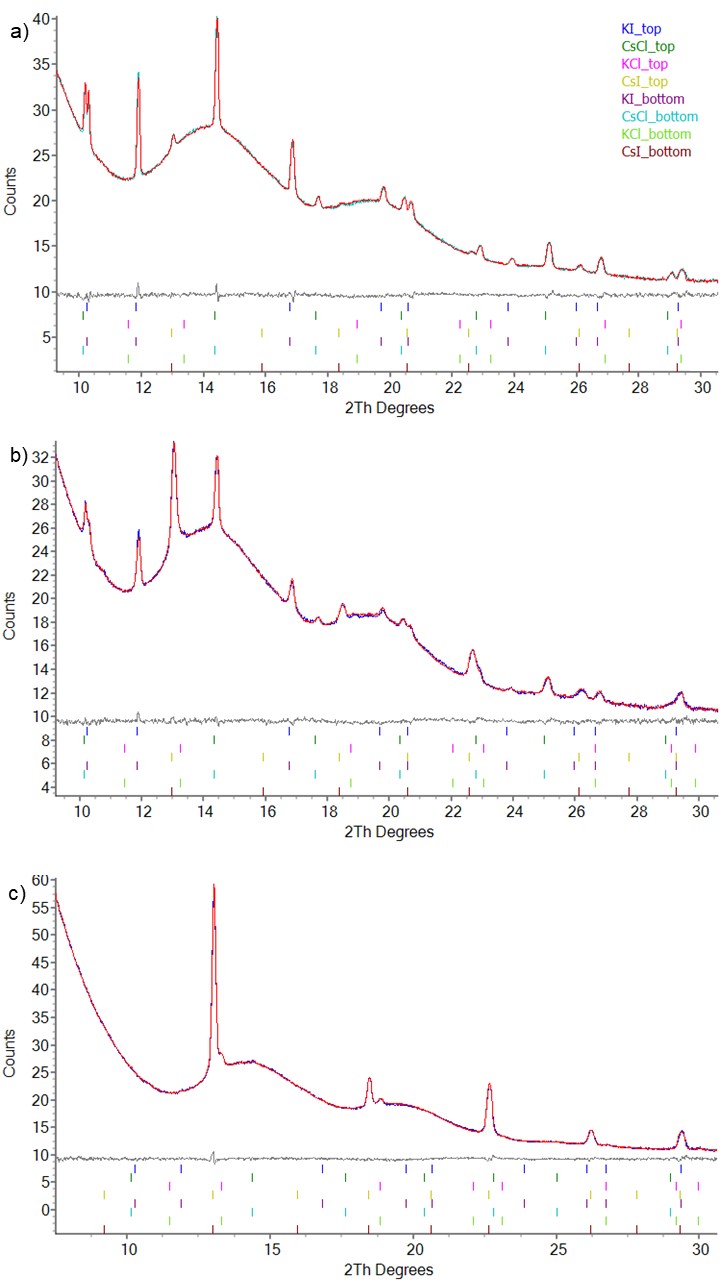


**Supplementary Figure 16**| Representative Rietveld plots for the sequential refinement of the inorganic metathesis reaction (Reaction I): a) scan 010, start of the experiment; b) scan 028, halfway the experiment; c) scan 098, end of the experiment. Calculated profile (red line) against experimental data: green line for plot a, blue line for plot b and c) and difference pattern (grey line). Peak marks are shown for all phases twice, once for the inner scattering ring, and once for the outer scattering ring – a legend with the corresponding colour for each phase is shown in the top right corner of (a).

## **Supplementary Note 5.3| Reaction IIIa:** **theophylline (tp) + benzamide (ba) = 1:1 tp:ba co-crystal Form I**

Lattice parameters, scaling factors and CS parameters were refined individually for all scans. The scale factors of theophylline and benzamide were constrained to keep their stoichiometric ratio as 1:1. Each scan was normalized using the scale parameter of the empirical background at the end of the relative Rietveld refinement as a normalization factor. The sequential Rietveld refinement was then performed again as described. The plots presented here are the output of the sequential Rietveld refinement performed on the dataset after this normalization step. The normalization helped eliminating an artifact from the CS versus milling time plots, *i.e.* a sudden step in the CS of Form I near the end of the time interval investigated. **Supplementary Figure 17** shows plots of the *R_wp_* and *χ^2^* for all the scans as obtained from the sequential Rietveld refinement. **Supplementary Figure 18** shows representative Rietveld plots of three scans at (a) the start of the experiment, (b) the midpoint of the experiment, and (c) the end of the experiment.


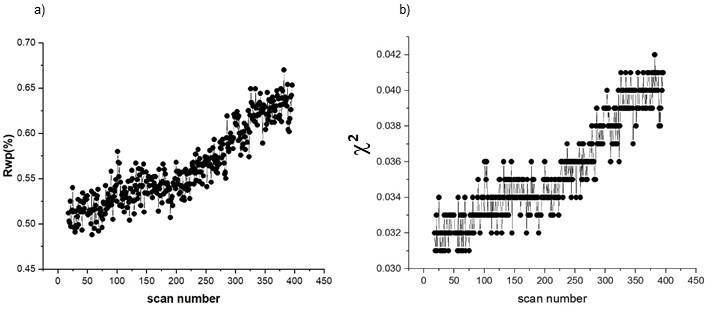


**Supplementary Figure 17|** Fit statistics for Rietveld refinement of the organic cocrystallisation between theophylline (tp) and benzamide (ba) to produce Form I of the 1:1 tp:ba cocrystal (Reaction IIIa), showing R_wp_ (a; Supplementary Equation 13) and χ^2^ (b; Supplementary Equation 14) for all the scans as obtained from the sequential Rietveld refinement.


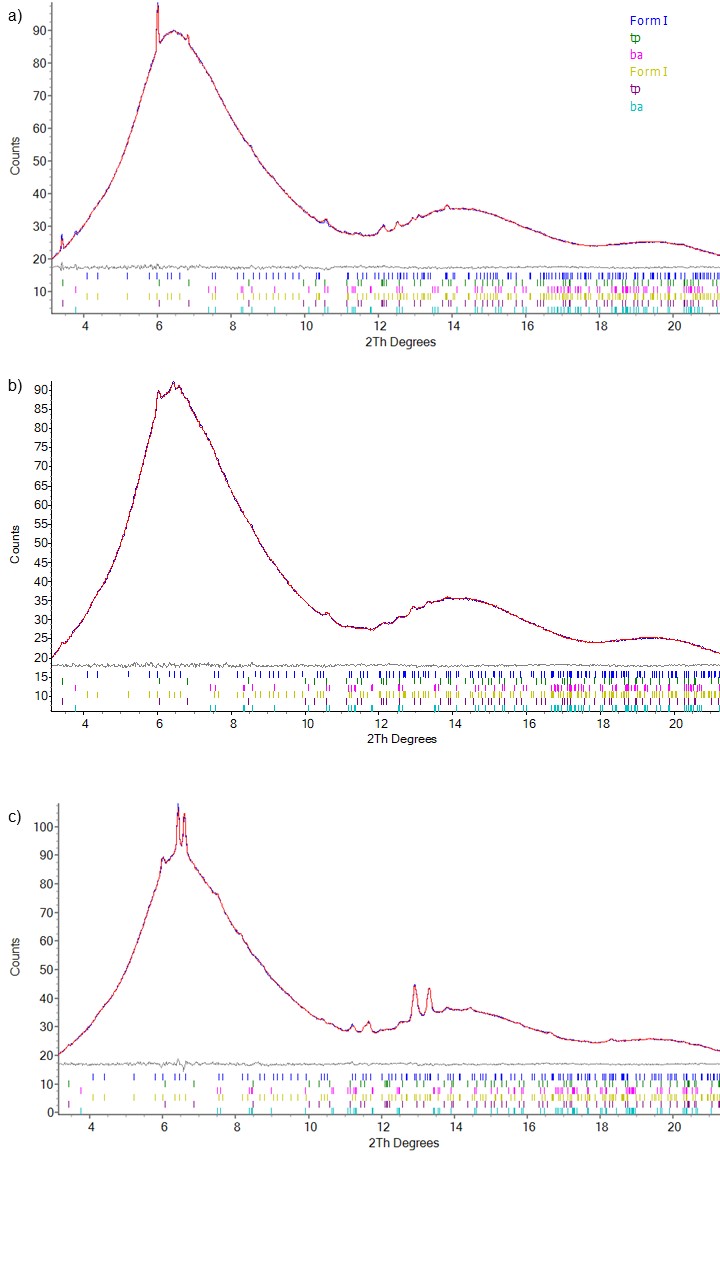


**Supplementary Figure 18**| Representative Rietveld plots for the sequential refinement of the organic cocrystallisation between theophylline (tp) and benzamide (ba) to produce Form I of the 1:1 tp:ba co-crystal (Reaction IIIa) : a) scan 005, start of the experiment; b) scan 148, halfway the experiment; c) scan 390, end of the experiment. calculated profile (red line) against experimental data (blue line) and difference pattern (grey line). Peak marks are shown for all phases twice, once for the inner scattering ring, and once for the outer scattering ring – a legend with the corresponding colour for each phase is shown in the top right corner of (a).

## **Supplementary Note 5.4| Reaction IIIb: theophylline (tp) + benzamide (ba) = 1:1 tp:ba co-crystal Form II**

Scaling factors and CS parameters were refined individually for all scans. Lattice parameters were all kept fixed. The sum of the scale factors of theophylline and monohydrate theophylline and the scale factor of benzamide were constrained to keep their stoichiometric ration as 1:1. **Supplementary Figure 19** is a cascade plot of the experimental patterns 005 to 018 showing the transformation of tp into monohydrate tp. **Supplementary Figure 20** shows plots of the *R_wp_* and *χ^2^* for all the scans as obtained from the sequential Rietveld refinement. **Supplementary Figure 21** shows representative Rietveld plots of five scans at (a) the start of the experiment, (b) the midpoint of the transformation of tp to monohydrate tp, (c) the end of the transformation of tp to monohydrate tp, (d) the midpoint of the cocrystallization of Form I, and (e) the end of the experiment.


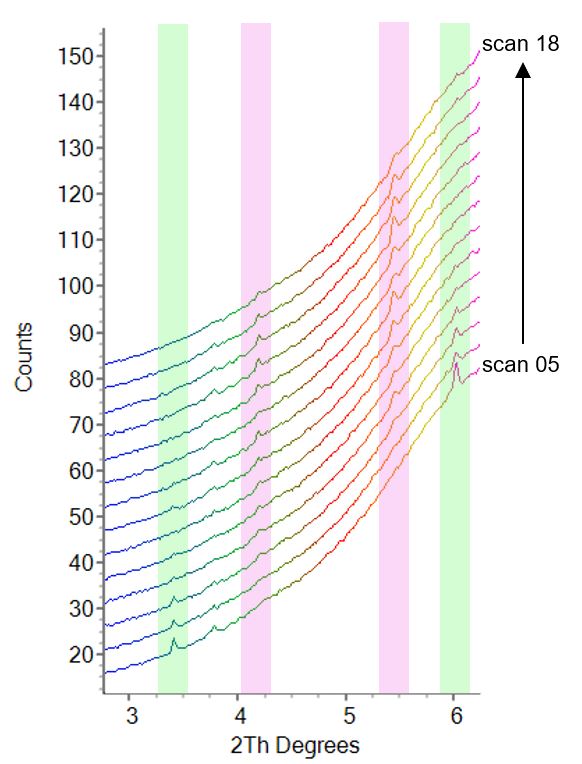


**Supplementary Figure 19**| Experimental diffraction pattern 005 to 018 of Reaction III under LAG conditions with water (theophylline (tp) + benzamide (ba) → 1:1 tp:ba co-crystal Form II), showing the transformation of tp (diffraction peaks highlighted in green) into monohydrate tp (diffraction peaks highlighted in purple).


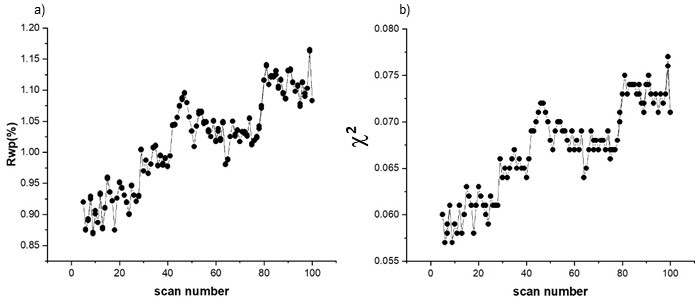


**Supplementary Figure 20**| Fit statistics for Rietveld QPA refinement of the organic cocrystallisation between theophylline (tp) and benzamide (ba) to produce Form II of the 1:1 tp:ba co-crystal (Reaction IIIb) showing R_wp_ (a; Supplementary Equation 13) and χ^2^ (b; Supplementary Equation 14) for all the scans as obtained from the sequential Rietveld refinement.


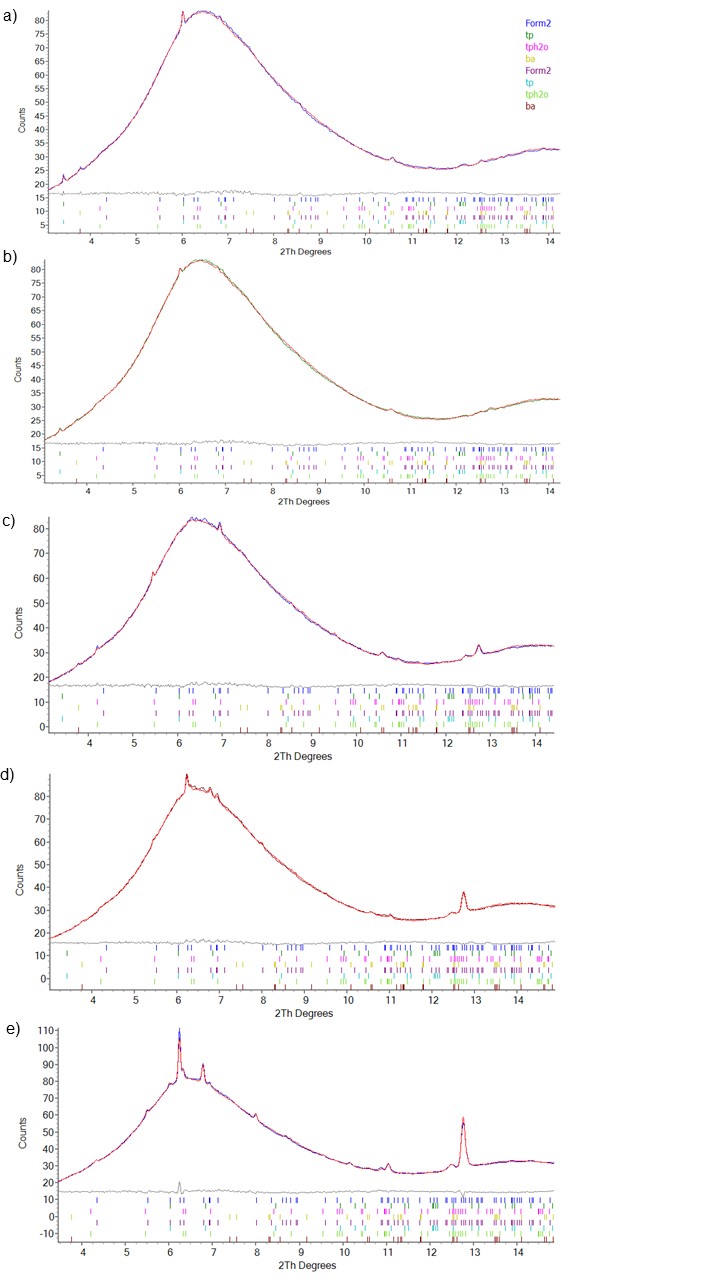


**Supplementary Figure 21**| Representative Rietveld plots for the QPA sequential refinement of the organic cocrystallisation between theophylline (tp) and benzamide (ba) to produce Form II of the 1:1 tp:ba co-crystal (Reaction IIIb): a) scan 005, start of the experiment; b) scan 006, halfway the transformation of tp to monohydrate tp; c) scan 016, end of the transformation of tp to monohydrate tp; d) scan 023, halfway the cocrystallization of Form I; e) scan 093, end of the experiment. Calculated profile (red line) against experimental data (blue line in plot a, c, e; green line in plot b; purple line in plot d) and difference pattern (grey line). Peak marks are shown for all phases twice, once for the inner scattering ring, and once for the outer scattering ring – a legend with the corresponding colour for each phase is shown in the top right corner of (a).

The CS values of Form II were taken from the sequential Rietveld QPA described above. To reduce the ESDs on the CS values for the co-formers, a fixed parametric approach was used for the scale factors.^10^ For each compound, a Boltzmann sigmoidal function was fit to the scale factor values as obtained from the sequential Rietveld QPA (see **Supplementary Figure 22**). The sequential Rietveld refinement was then performed with all scale factors constrained to the relative sigmoidal function. The CS values for the co-formers shown in **Supplementary Figure 22** were taken from this sequential Rietveld analysis. **Supplementary Figure 23** shows plots of the *R_wp_* and *χ^2^* for all the scans as obtained from the parametric sequential Rietveld refinement. **Supplementary Figure 24** shows representative Rietveld plots of four scans at (a) the end of the transformation of tp to monohydrate tp, (b) the midpoint of the cocrystallization of Form I, and (c) the end of the experiment.


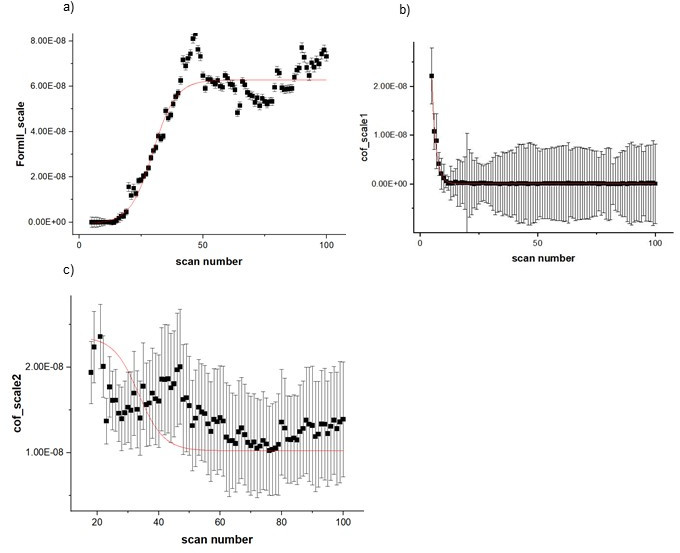


**Supplementary Figure 22**| Sigmoidal fits and functions used to constrain the scale factors of the product (a) and the coformers (b and c) in the parametric sequential Rietveld refinement for the microstructural analysis of the organic cocrystallisation between theophylline (tp) and benzamide (ba) to produce Form II of the 1:1 tp:ba co-crystal. a) y = FormII_scale = 6.28 * 10^-8^ + ( ( 6.34 * 10^-8^ ) ) / (1 + Exp ( ( scan_number -29.8 ) / 4.6 ) ) ; R^2^ = 0.95. b) cof_scale1 = 6 * 10^-11^ + ( ( 1 * 10^-6^ ) ) / (1 + Exp ( ( scan_number + 3) / 1.8 ) ) ; R^2^ = 0.97. c) cof_scale2 = 1 * 10^-8^ + ((1 * 10^-8^) / (1 + Exp ( ( scan_number – 33 ) / 4 ) ) ; R^2^ = -0.92.


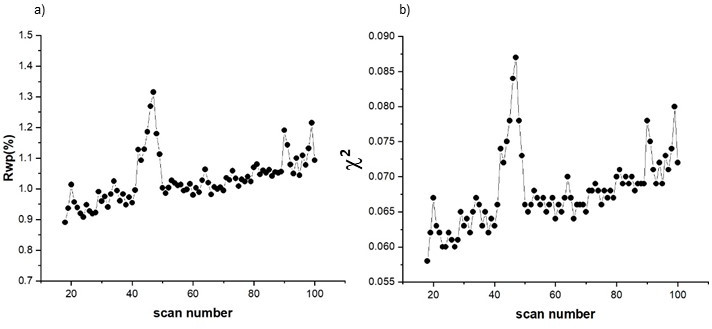


**Supplementary Figure 23**| Fit statistics for Rietveld refinement for the microstructural analysis of the coformers in the organic cocrystallisation between monohydrate theophylline (tp) and benzamide (ba) to produce Form II of the 1:1 tp:ba co-crystal.showing R_wp_ (a; Supplementary Equation 13) and χ^2^ (b; Supplementary Equation 14) for all the scans as obtained from the sequential parametric refinement.


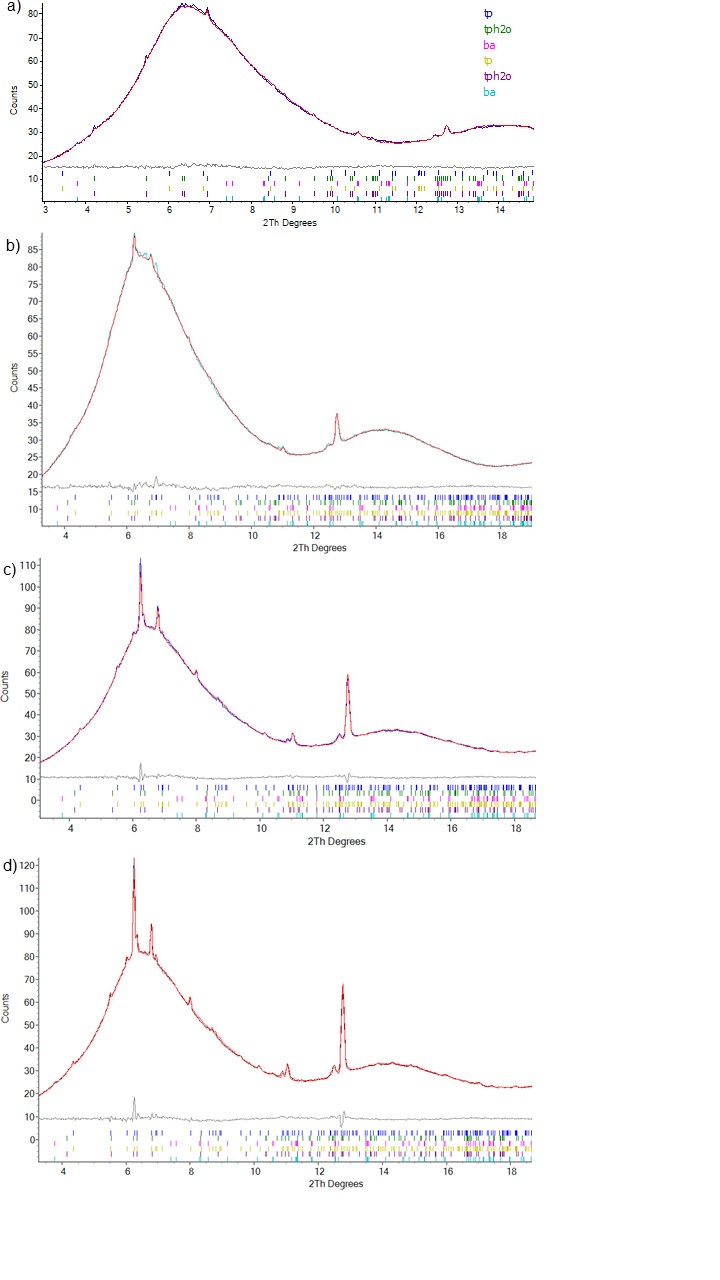


**Supplementary Figure 24**| Representative Rietveld plots for the microstructural analysis of theophylline (tp) and benzamide (ba) in the organic cocrystallisation of Form II of the 1:1 tp:ba co-crystal (Reaction IIIb) via sequential refinement: a) scan 016, end of the transformation of tp to monohydrate tp; b) scan 023, halfway the cocrystallization of Form I; c) scan 093, end of the experiment. Scan 047 has been added to allow for a visual evaluation of the fit quality given the relatively higher values of its R_wp_ and χ^2^ shown in Supplementary Figure 23. Calculated profile (red line) against experimental data (blue line in plot a and c; pale blue line in plot b; brown line in plot d) and difference pattern (grey line). Peak marks are shown for all phases twice, once for the inner scattering ring, and once for the outer scattering ring – a legend with the corresponding colour for each phase is shown in the top right corner of (a).

## **Supplementary note 5.5| Reaction IV - Covalent disulfide exchange reaction**

Lattice parameters, scaling factors and CS parameters were refined individually for all scans. The scale factors of bis(2-nitrophenyl) disulfide and bis(4-chlorophenyl) disulfide (2NO_2_PhS and 4ClPhS, respectively) were constrained to keep their stoichiometric ratio equal to 1:1. **Supplementary Figure 25** shows plots of the *R_wp_* and *χ^2^* for all the scans as obtained from the sequential Rietveld refinement. **Supplementary Figure 26** shows representative Rietveld plots of three scans at (a) the start of the experiment, (b) the midpoint of the experiment, and (c) the end of the experiment.


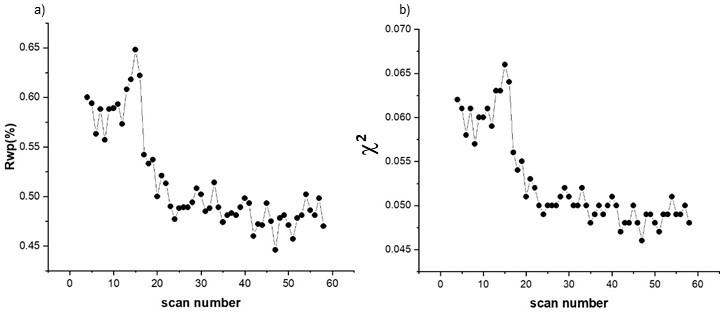


**Supplementary Figure 25**| Fit statistics for Rietveld refinement for the covalent disulfide exchange reaction (Reaction IV).showing R_wp_ (a; Supplementary Equation 13) and χ^2^ (b; Supplementary Equation 14) for all the scans as obtained from the sequential QPA refinement.


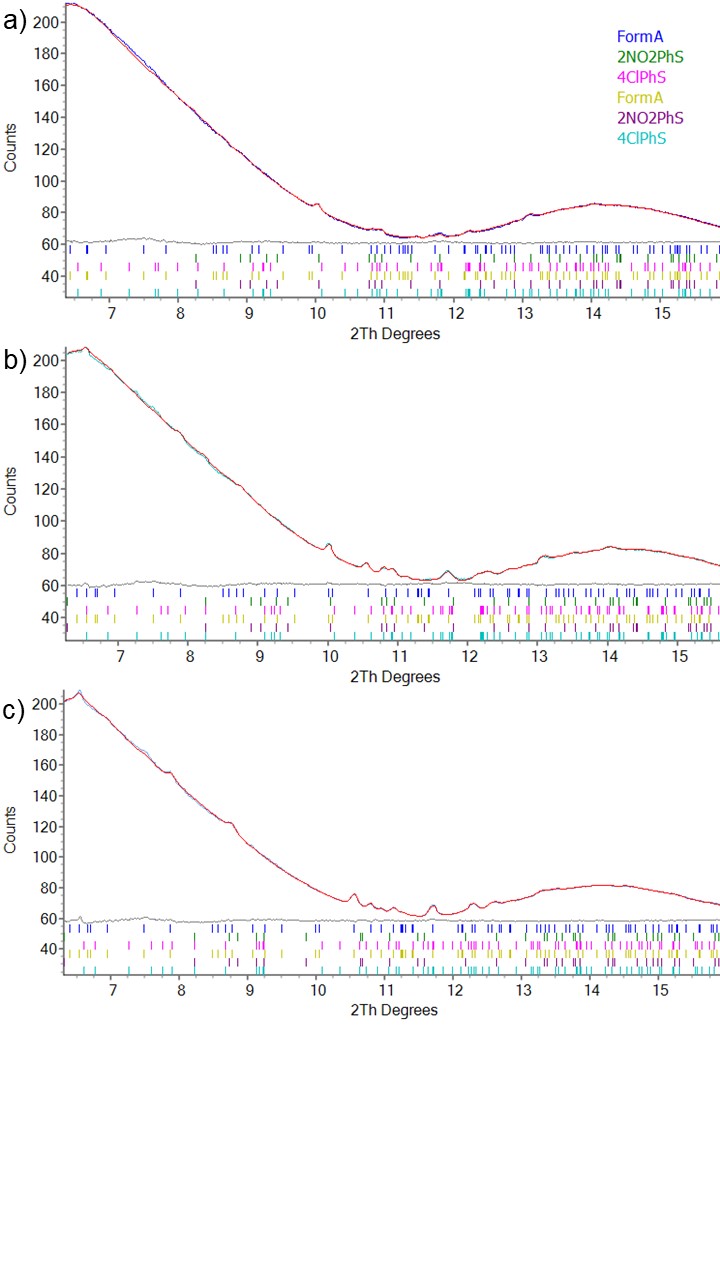


**Supplementary Figure 26**| Representative Rietveld plots for the QPA sequential refinement of the covalent disulfide exchange reaction (Reaction IV) between bis(2-nitrophenyl) disulfide and bis(4-chlorophenyl) disulfide (2NO2PhS and 4ClPhS, respectively) : a) scan 004, start of the experiment; b) scan 020, halfway exchange reaction; c) scan 056, end of the experiment. calculated profile (red line) against experimental data (blue line in plot a; pale blue line in plot b and c) and difference pattern (grey line). Peak marks are shown for all phases twice, once for the inner scattering ring, and once for the outer scattering ring – a legend with the corresponding colour for each phase is shown in the top right corner of (a).

To reduce the ESDs on the CS values for all compounds, a fixed parametric approach was used for the scale factors.^10^ For each compound, a Boltzmann sigmoidal function was fit to the scale factor values as obtained from the sequential Rietveld QPA (see **Supplementary Figure 27**). The sequential Rietveld refinement was then performed with all scale factors constrained to the relative sigmoidal function. The CS values for all compounds shown in **Figure 6** in the main text were taken from this sequential Rietveld analysis. **Supplementary Figure 28** shows plots of the *R_wp_* and *χ^2^* for all the scans as obtained from the parametric sequential Rietveld refinement. **Supplementary Figure 29** shows representative Rietveld plots of three scans at (a) the start of the experiment, (b) the midpoint of the experiment, and (c) the end of the experiment.


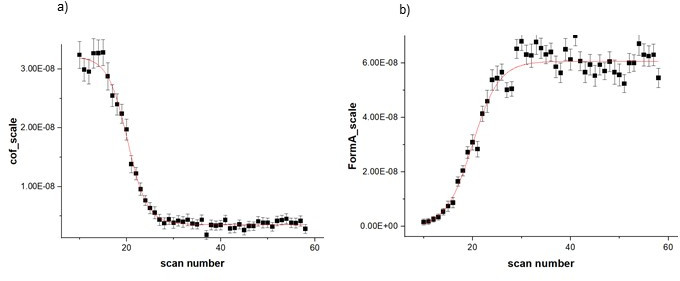


**Supplementary Figure 27**| Sigmoidal fits and functions used to constrain the scale factors of the reactants (a) and the product (b) in the parametric sequential Rietveld refinement for the microstructural analysis of the covalent disulfide exchange reaction (Reaction IV). a) cof_scale = (3.6 * 10^-9^ + ( ( 2.95 * 10^-8^ ) ) / (1 + Exp ( ( scan_number - 20.2) / 2.1 ) ) ; R^2^ = 0.99. b) FormA_scale = 6.06 * 10^-8^ + ( ( - 6.1 * 10^-8^ ) / (1 + Exp ( ( scan_number - 20) / 2.7 ) ) ; R^2^ = 0.98.


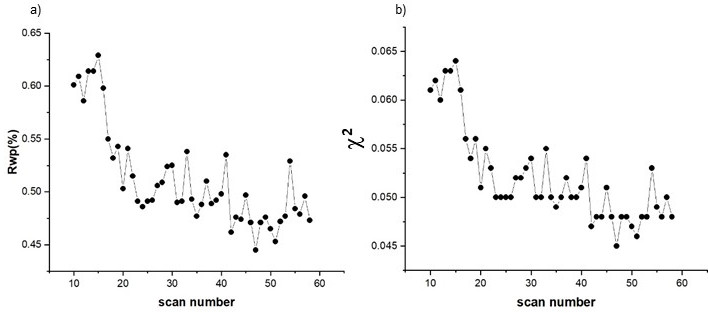


**Supplementary Figure 28**| Fit statistics for Rietveld refinement for the covalent disulfide exchange reaction (Reaction IV).showing R_wp_ (a; Supplementary Equation 13) and χ^2^ (b; Supplementary Equation 14) for all the scans as obtained from the sequential parametric refinement.


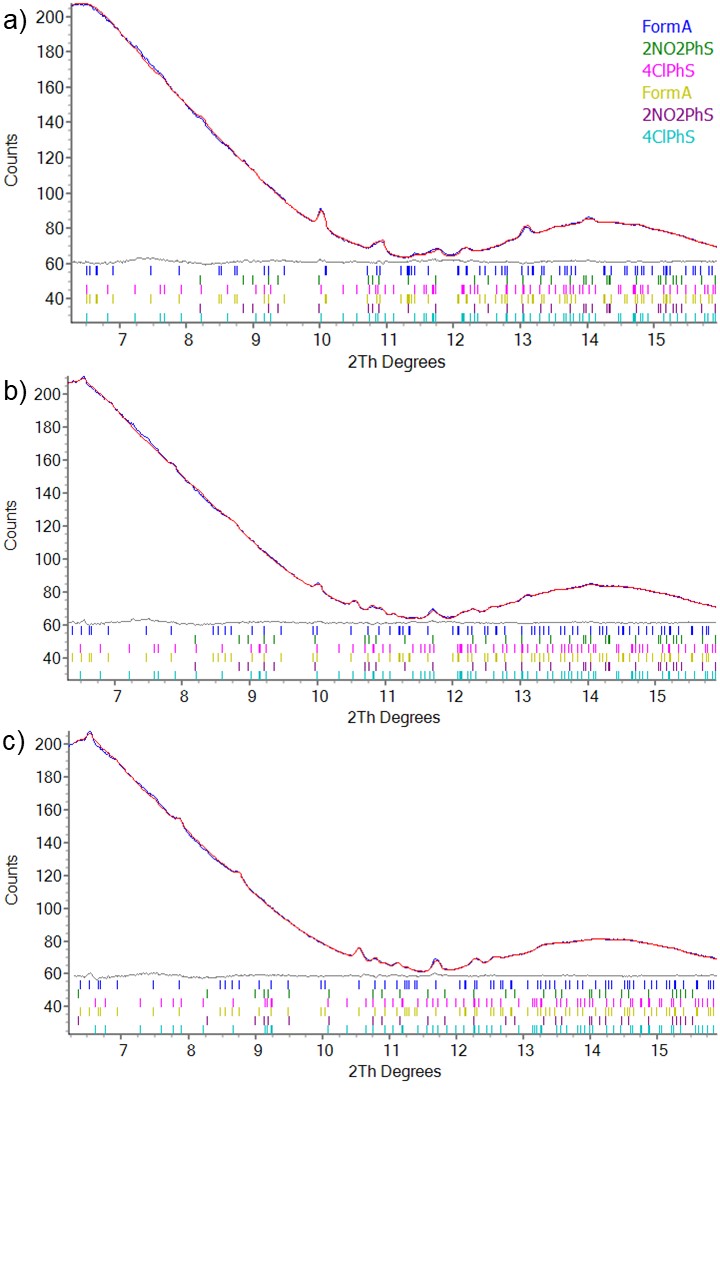


**Supplementary Figure 29**| Representative Rietveld plots for the microstructural analysis of the covalent disulfide exchange reaction (Reaction IV) between bis(2-nitrophenyl) disulfide and bis(4-chlorophenyl) disulfide (2NO2PhS and 4ClPhS, respectively) via sequential refinement: a) scan 004, start of the experiment; b) scan 020, halfway exchange reaction; c) scan 056, end of the experiment. Calculated profile (red line) against experimental data: blue line in plot a, pale blue line in plot b and c) and difference pattern (grey line). Peak marks are shown for all phases twice, once for the inner scattering ring, and once for the outer scattering ring – a legend with the corresponding colour for each phase is shown in the top right corner of (a).

## **Supplementary Note 5.6| Reaction IV – Determination of chemical composition of disulfide exchange by HPLC**

On completion of the experiment described in **Supplementary Note 4.4**, the jar was sampled in four areas as shown in **Supplementary Figure 30** to check for chemical composition homogeneity. Around 0.3 to 0.8 mg of powder were transferred to a HPLC vial as described in **Supplementary Table 1**. As shown in **Supplementary Figure 30** and **Supplementary Table 1**, the reaction went quantitatively to the heterodimer and was uniformly transformed.


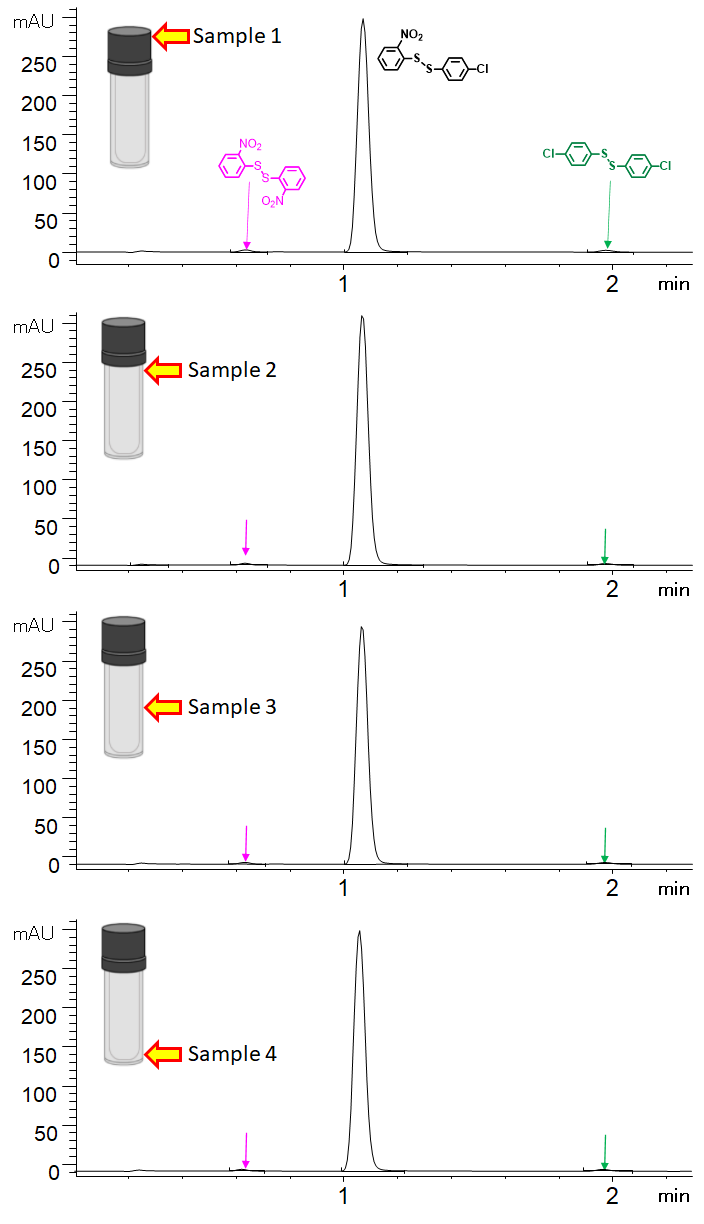


**Supplementary Figure 30**| HPLC chromatograms of the 4 samples taken from different locations in the grinding jar as indicated in the figure. The samples were taken after completion of the TRIS experiment described in Supplementary Note 4.4. See Supplementary table 1 for the corresponding chemical composition, demonstrating that the neat ball mill grinding reaction reached completion and was homogenously distributed in the grinding jar. The HPLC method is described in Supplementary note 1.3. The corresponding HPLC data is shown in Supplementary Table 1.

**Supplementary Table 1**| Composition of the 4 samples of the disulfide exchange experiments described in Supplementary Note 4.4 at 10 mg loading taken from the 1.8 mL grinding jar. All 4 samples show similar chemical composition demontrating that the ball mill neat grinding reaction was quantitative and that the chemical composition in the jar was homogenous. The HPLC method is described in Supplementary Note 1.3. HPLC chromatograms are shown in Supplementary Figure 30.


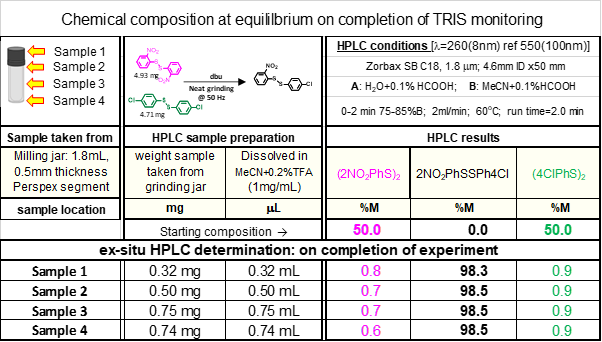


## **Supplementary Note 5.7| Improvement of crystal size and microstrain analysis calculations applied to Reaction I - TRIS Monitoring of Inorganic Solid Mechanochemistry**

We here show an alternative data analysis via sequential Rietveld refinement including microstructural parameters for crystal size (CS) and microstrain (also called just strain) ε_0_ for the model inorganic metathesis reaction KI + CsCl $\boldsymbol{\to}$ KCl + CsI (Reaction I), **Supplementary Figure 31**.


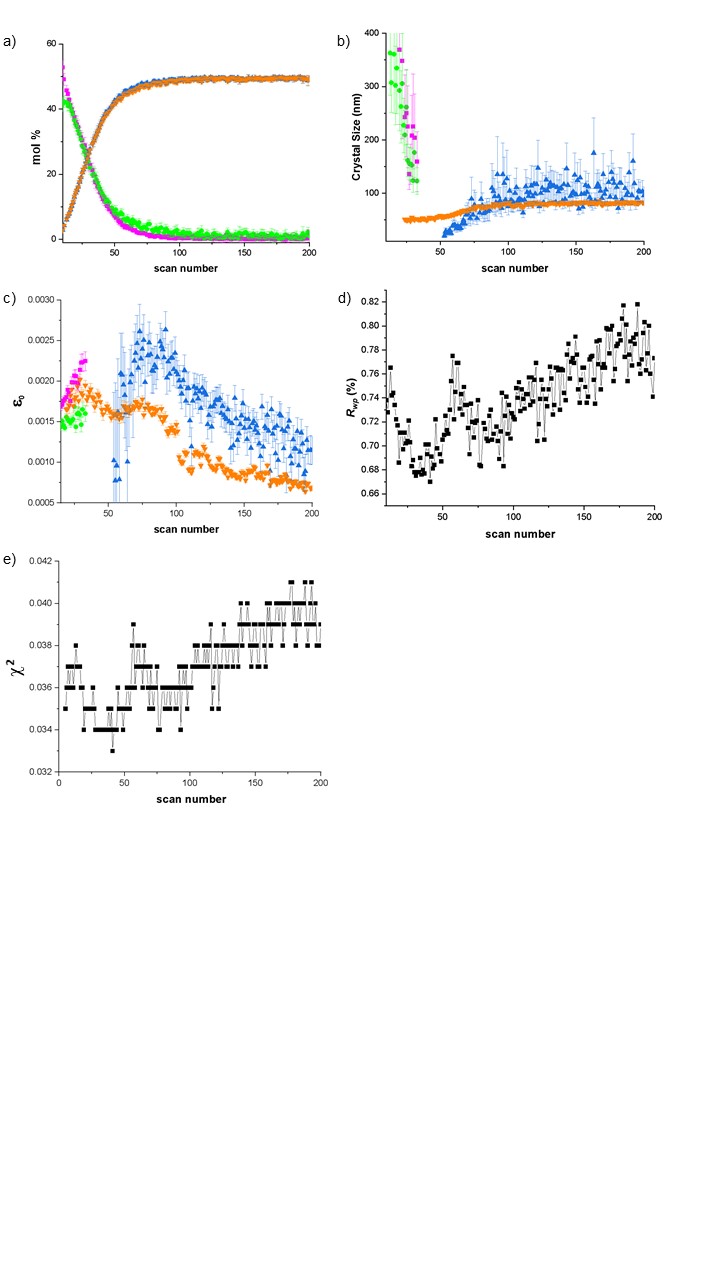


**Supplementary Figure 31**| Output plots of the sequential Rietveld analysis including microstructural parameters for crystal size and (micro)strain for the model inorganic metathesis reaction KI + CsCl → KCl + CsI (Reaction I). a) Quantitative phase analysis for the inorganic metathesis reaction obtained by Rietveld refinement, showing the consumption of KI (pink) and CsCl (green), with simultaneous formation of KCl (blue) and CsI (orange) with relative EDS bars. (b) Scherrer crystallite size obtained for KI (pink), CsCl (green), KCl (blue) and CsI (orange) with relative ESD bars are shown for phases more abundant than 20 M%. (c) Microstrain ε_0_ obtained for KI (pink), CsCl (green), KCl (blue) and CsI (orange) with relative ESD bars are shown for phases more abundant than 20 M%. (d,e) Goodness of fit parameters *R_wp_* (Supplementary Equation 13) and *χ^2^* (“chi square”, Supplementary Equation 14) for all the scans as obtained from the sequential Rietveld refinement for the inorganic metathesis reaction (Reaction I).

In general, the crystallinity of a sample is determined by its crystal size and strain. (Micro)strain ε_0_ is the nonuniform variation in the unit cell and interplanar spacings *d* caused by local distortion of lattice planes. It is defined as Δ(*d*)/*d,* and it is adimensional. The distortion is the direct consequence of defects such as vacancies, substitutions, or dislocations. The diffraction angle $\theta$ of radiation with wavelength $\lambda$ depends on the interplanar spacing in agreement with Bragg’s Law:

$2\theta=2arcsin\left( \frac{\lambda}{2d} \right)$ Supplementary Equation 16

The more defects, the less constant is the value of *d*, and hence the broader will be the diffraction peaks (**Supplementary Figure 32**).


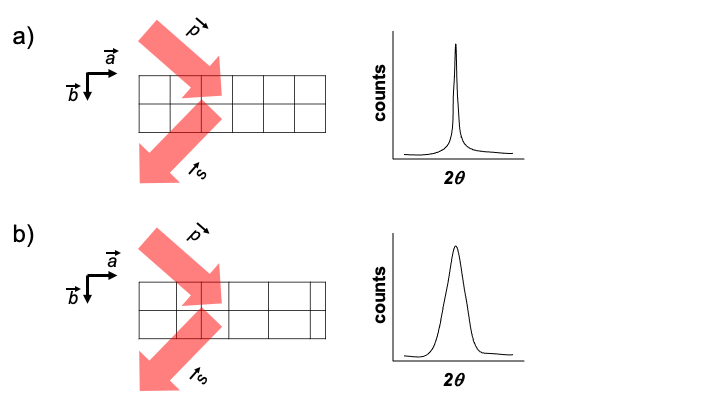


**Supplementary Figure 32** | Schematic representation of the effect of non-uniform lattice distortion on the diffraction peak broadening: $\vec{a}$, $\vec{b}$ are the unit cell axes, represented by the rectangular grid; $\vec{p}$ and $\vec{s}$ represents the incident and diffracted radiation respectively. a) an undistorted lattice (left) yielding narrow diffraction peaks (right); b) non uniform distortion of the lattice along the $\vec{a}$ crystallographic direction, i.e. a distribution of the interplanar d spacings around an average $\vec{a}$ values (left), causing a broadening of the diffraction peaks.

Lattice parameters, scaling factors and CS parameters were refined individually for all scans. Each scan was normalized using the scale parameter of the empirical background at the end of the relative Rietveld refinement as a normalization factor. The sequential Rietveld refinement was then performed again as described in **Supplementary Note 5.2**. Size and strain for all four phases were refined with a Lorentzian and Gaussian function, respectively. A Lorentzian function was convoluted for each phase, with a single isotropic CS parameter related to Lorentzian peak width $\Gamma_{L}$ as in the Scherrer Equation (**Supplementary Equations 11 and 12**) described in **Supplementary Note 5.1**. A Gaussian function was convoluted for each phase, with a single isotropic strain parameter ε_0_ related to $\Gamma_{G}$ (the FWHM of a Gaussian curve) as in the following equation:^4^

$e_{0}=\frac{\Gamma_{G}\left( 2\theta\right)}{4\left( tan\theta\right)}$ Supplementary Equation 17

Thus, the strain contribution to peak broadening depends on the diffraction angle *θ* in a different way from CS (See **Supplementary Note 5.1**), which makes deconvolution of sample CS and strain contributions possible. The top and bottom phases of the same compound were constrained to have the same CS and strain parameters. Lattice parameters, scaling factors and microstructural parameters were refined individually for all scans. The scale factors of KCl and CsI were constrained to keep their stoichiometric ratio as 1:1. **Supplementary Figure 33** shows representative Rietveld plots of three scans at: the start of the experiment; the midpoint of the experiment; the end of the experiment. The CS and ε_0_ plots (i.e. plots of strain, **Supplementary Equation 17**) of **Supplementary Figure 31** suggest that while the CS of the product phases reaches a plateau, the strain keeps decreasing to the end of the recorded dataset. In other words: prolonged milling increases the products crystallinity in terms of fewer lattice defects (continued decrease in strain, **Supplementary Figure 31c** and **Supplementary Equation 17**) rather than further crystal growth (plateau in CS, **Supplementary Figure 31b**).

As a general comment, if the aim is that of separating CS and strain contributions it is advisable to extend the 2*θ* range monitored. As the strain contribution is more significant at higher angle, a larger range will help minimizing the ESDs on size and strain parameters.


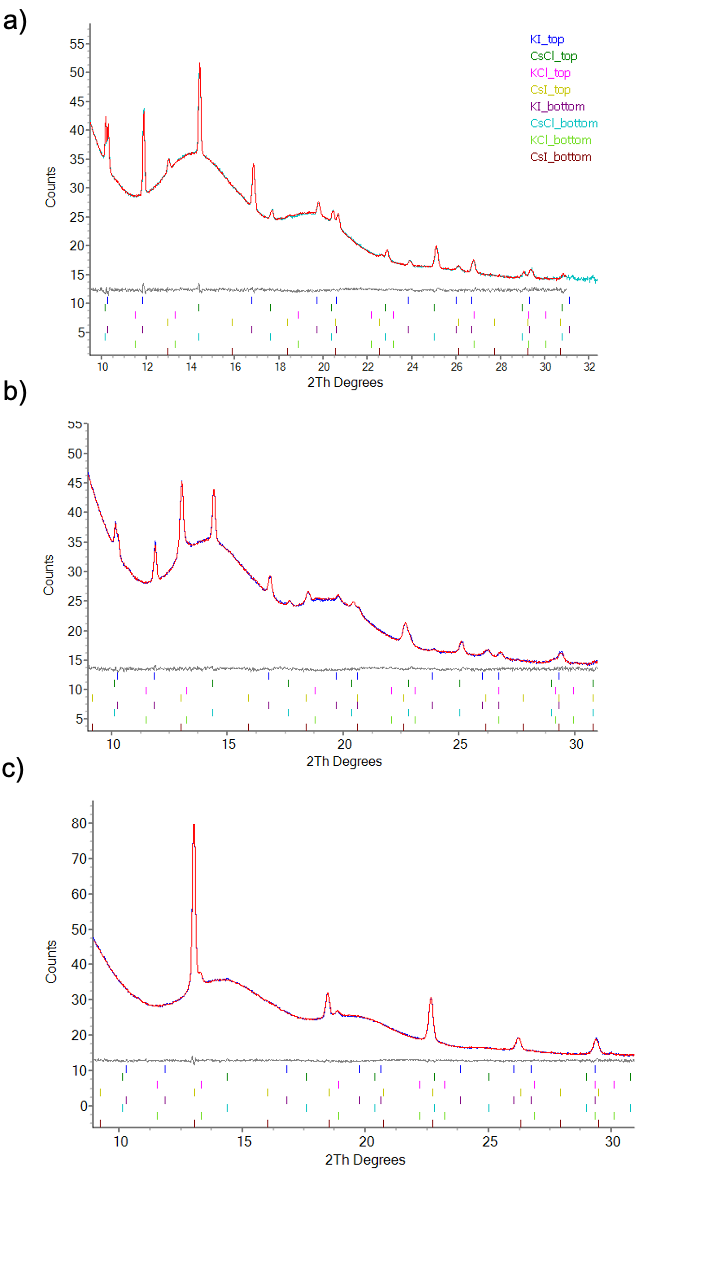


**Supplementary figure 33** | Representative Rietveld plots for the sequential refinement of the inorganic metathesis reaction (Reaction I): a) scan 010, start of the experiment; b) scan 028, halfway the experiment; c) scan 098, end of the experiment. Calculated profile (red line) against experimental data (green line for plot a, blue line for plot b and c) and difference pattern (grey line). Peak marks are shown for all phases twice, once for the inner scattering ring (“top” phases), and once for the outer scattering ring (“bottom” phases) – a legend with the corresponding colour for each phase is shown in the top right corner of (a).

# **SUPPLEMENTARY NOTE 6| EX SITU ANALYSIS OF COMPLETED SYNCHROTRON EXPERIMENTS**

## **Supplementary note 6.1| Introduction**

We present here the analysis of the completed in-situ experiments for Reactions I, II and III. The analysis includes:

1) Photographs of the disassembled 3-component jar.

2) Distribution by the weight of the powder in the 3 components of the jar and the ball.

3) The distribution of the powder is classified as “loosened” which should equate to free-flowing powder and “scraped” which equated to powder that adheres to the walls of the jar.

4) Rietveld refinement processing of XRPD scans of powder removed from jar at different locations, some loosened, some scraped, other combined because there is not enough powder to run them individually. Here we obtain phase composition and Scherrer crystal size of starting materials and products.

The supporting data will be presented in the following sections:

Supplementary Note 6.3: Reaction I (NG at 50 Hz to equilibrium: 8.0 mm ss ball- 70 mg payload)

Supplementary Note 6.4: Reaction II (ILAG at 50 Hz to equilibrium: 8.0 mm ss ball- 70 mg payload)

Supplementary Note 6.5: Reaction III (NG at 50 Hz to equilibrium: 7.0 mm ss ball- 60 mg payload)

Supplementary Note 6.6: Reaction III (LAG/9μL water at 50 Hz to equilibrium: 7.0 mm ss ball- 60 mg payload)

## **Supplementary Note 6.2| Overall conclusions**

## **Supplementary Note 6.2.1| Powder distribution in the milling jar**

For the more friable inorganic powders (Reactions I and II), the majority of material remains free flowing (loose) by the end of the reaction. In contrast, the softer organic solids (Reactions III) appear to stick to surfaces within the milling jar over the course of the ball mill grinding. We expect this rheological difference between inorganic and organic powders to be a general for ball milling of powders, and must be considered when interpreting TRIS XRPD data. In the loose powder (Reaction I), the phase composition and crystallite size are consistent over samples taken from across the milling jar, reflecting the high degree of mixing that occurs during ball mill grinding. In contrast, the extent of cocrystallisation in the organic system (Reaction III) depended critically on the sampling location of the powder. Powder which was stuck to the Perspex walls or jar ends comprised a high degree of reaction product (ca. 90-95%), whereas loose powder contained only ca. 35-40% product for neat grinding transformations. We note however that starting materials appeared to disproportionate between the free flowing and stuck powder, thereby changing the effective composition (non-stoichiometric) of the free-flowing powder. The homogeneity was improved by liquid assisted grinding. This is consistent with earlier observations of sample location-dependent reaction yields in the ball mill grinding of organic cocrystal formation.^11^ We therefore suggest that care should be taken when interpreting reaction kinetics obtained by TRIS XRPD, where the exact composition of powder at one location may not be entirely representative of the bulk powder at any given time.

**Supplementary Note 6.2.2| Comparison Between In Situ and Ex Situ Rietveld Analyses**

*Ex situ* data Rietveld analyses were compared with Rietveld outputs from the last scan of the relative in situ dataset. The Rietveld analysis strategy of in situ data is discussed in **Supplementary Note 5.1**.

Ex situ XRD data were collected two days after milling experiments were performed. Data were collected using a Bruker D8 Advanced diffractometer equipped with a CuKα X-ray tube in Bragg-Brentano geometry over a 5° to 45° range with a resolution of 0.2°/step, with a collection time of 0.35 s/step (total *ca.* 9 mins). A LaB_6_ 660d NIST standard^12^ was used to model the instrumental contribution to peak broadening using a fundamental parameters approach^13^ with Topas V6.^7^ Lattice parameters, scaling factors, and no other structural parameter were refined for each phase in the quantitative Rietveld refinements of the *ex situ* data. A Chebyshev function with seven parameters was used to fit the background. Sample contribution to peak broadening was assumed to be related to CS only (see **Supplementary Note 5.1**). The same approach was used for Reaction I: *ex situ* data included only one diffraction peak for each of the product phases which would give a near 100% negative correlation of the strain parameter with the CS parameter. A Lorentzian peak shape for CS was found to fit better than a Gaussian one. To minimise the correlations and the ESD, and to facilitate comparison with *in situ* data, CS contribution to peak broadening was modelled as isotropic. We remind that the ESD from the Rietveld calculation has no bearing on the precision or accuracy, but is only related to the mathematical fit of the model.^9^ Concerning the accuracy of size estimates, it is known that for a typical laboratory X-ray diffraction instrument the Scherrer analysis provides sensitivity to crystallite size in the 1–100 nm range;^8^ the upper limit is set by the instrumental broadening. Thus, the smaller the crystal size, the less the Scherrer CS size value is affected by the instrumental broadening definition. For example, for Reaction IV, a Scherrer crystal size of ~100 nm can vary up to 30% depending on the way the fundamental parameters are used to fit the LaB_6_ 660d NIST standard. For example, depending on whether size and strain contributions of the NIST standard itself are assumed to be zero or are allowed to contribute to the LaB_6_ peak shape, and how some fundamental parameters (especially those affecting the peak broadening at low angle where the NIST standard has no peaks, like divergence slit and soller slits parameters)^13^ are set and/or refined. However, if the crystal size is ~60 nm, this variation is no more than 10%. In other words, the smaller the crystal size, the more reliable the number. Thus, considering the very large accuracy error for a value of 170 nm, we must consider critically the CS values of Form II of the co-crystal (**Supplementary Figure 37** e and d tables). The apparent decrease of ~50 nm from *in situ* to *ex situ* may be due to inherent accuracy limits. While we can be confident about a difference larger than the reported ESD in the same dataset, this is not advisable when comparing two samples collected with two very different instruments, even when NIST standards are used to model the relative instrumental contributions. That said, we do not exclude that the crystallinity of this phase may have changed over the 48h lag between the *in situ* measurement and the *ex situ* one. To support this last statement, it is interesting that in Reaction I (**Supplementary Figure 34**), CsI must have grown in crystal size considering that KCl shows not to have changed CS over the 48 hours lag between the two measurements. We note that strain contribution was account for in Reaction I *in situ* data refinement to make this comparison. In terms of phase quantification, our alignment strategy maximized the signal of the powder stuck to the jar wall. However, we note that while starting materials appeared to disproportionate between the free flowing and stuck powder in Reaction III (**Supplementary Figures 36 and 37**), the free-flowing powder represents a very small fraction of the total powder. **Supplementary Figure 38** shows some representative Rietveld plots for the ex-situ data analysed.

## **Supplementary Note 6.3| Reaction I (NG at 50 Hz to equilibrium: 8.0 mm ss ball- 70 mg payload)**

| **** |  |
| --- | --- |

**Supplementary Figure 34|** Outcome of Reaction I at synchrotron (NG at 50 Hz using a 3-segment grinding jar: 8.0 mm ss ball- 70 mg payload a) left: Reaction scheme. Grinding conditions are depicted using pictograms;^14^ right: exploded diagram of grinding jar. b) tabulation of experimental parameters and pictures of jars opened on completion of milling. c) left: tabulation and graphical representation of distribution of powder on top & bottom caps, Perspex clear segment & ss ball; right: depiction where samples were taken from jar for analysis; d) tabulation of phase composition and Scherrer size obtained from ex situ XRPD analysis from 4 different location in the grinding jar as in c); e) tabulation of phase composition and Scherrer size using the last synchrotron in situ XRPD file: Please note that microstrain e0 was included in the calculations.

## **Supplementary Note 6.4| Reaction II (ILAG at 50 Hz to equilibrium: 8.0 mm ss ball- 70 mg payload)**

| **** |  |
| --- | --- |

**Supplementary Figure 35**| Outcome of Reaction II at synchrotron (Ionic Liquid assisted grinding (ILAG) with 17μL DMF/0.1%M NH_4_NO_3_) at 50 Hz using a 3 component grinding jar: 8.0 mm ss ball- 70 mg payload a) left: reaction scheme. Grinding conditions are depicted using pictograms;^14^ right: exploded diagram of jar. b) tabulation of experimental parameters, photo of jars opened on completion of milling and sketch where samples were taken for analysis. c) Tabulation and graphical representation of the distribution of powder on top & bottom caps, Perspex clear segment & ss ball; d) Rietveld refinement cannot be performed on the 4 ex-situ XRPD scans, as the documented structure of ZIF-8, has shown on in-situ experiments not to be correct.

## **Supplementary Note 6.5| Reaction III (NG at 50 Hz to equilibrium: 7.0 mm ss ball- 60 mg payload)**

| **** |  |
| --- | --- |

**Supplementary Figure 36|** Outcome of Reaction III at synchrotron (NG at 50 Hz using a 3 part grinding jar: 7.0 mm ss ball- 60 mg payload a) left: reaction scheme. Grinding conditions are depicted using pictograms;^14^ right: exploded diagram of jar. b) tabulation of experimental parameters, photo of jars opened on completion of milling. c) left: tabulation and graphical representation of distribution of powder on top & bottom caps, Perspex clear segment & ss ball; right: depiction where samples were taken from jar for analysis; d) tabulation of phase composition and Scherrer size obtained from ex situ XRPD analysis from 4 different locations in the grinding jar as in c); e) tabulation of phase composition and Scherrer size using the last synchrotron in situ XRPD file:

## **Supplementary Note 6.6| Reaction III (LAG/9μL water at 50 Hz to equilibrium: 7.0 mm ss ball- 60 mg payload)**

| **8** |  |
| --- | --- |

**Supplementary Figure 37|** Outcome of Reaction III at synchrotron (LAG/9μL water at 50 Hz using a 3 part grinding jar: 7.0 mm ss ball- 60 mg payload a) left: reaction scheme. Grinding conditions are depicted using pictograms;^14^ right: exploded diagram of jar. b) tabulation of experimental parameters, photo of jars opened on completion of milling. c) left: tabulation and graphical representation of distribution of powder on top & bottom caps, Perspex clear segment & ss ball; right: depiction where samples were taken from jar for analysis; d) tabulation of phase composition and Scherrer size obtained from ex situ XRPD analysis from 6 different locations in the grinding jar as in c); e) tabulation of phase composition and Scherrer size using the last synchrotron in situ XRPD file.


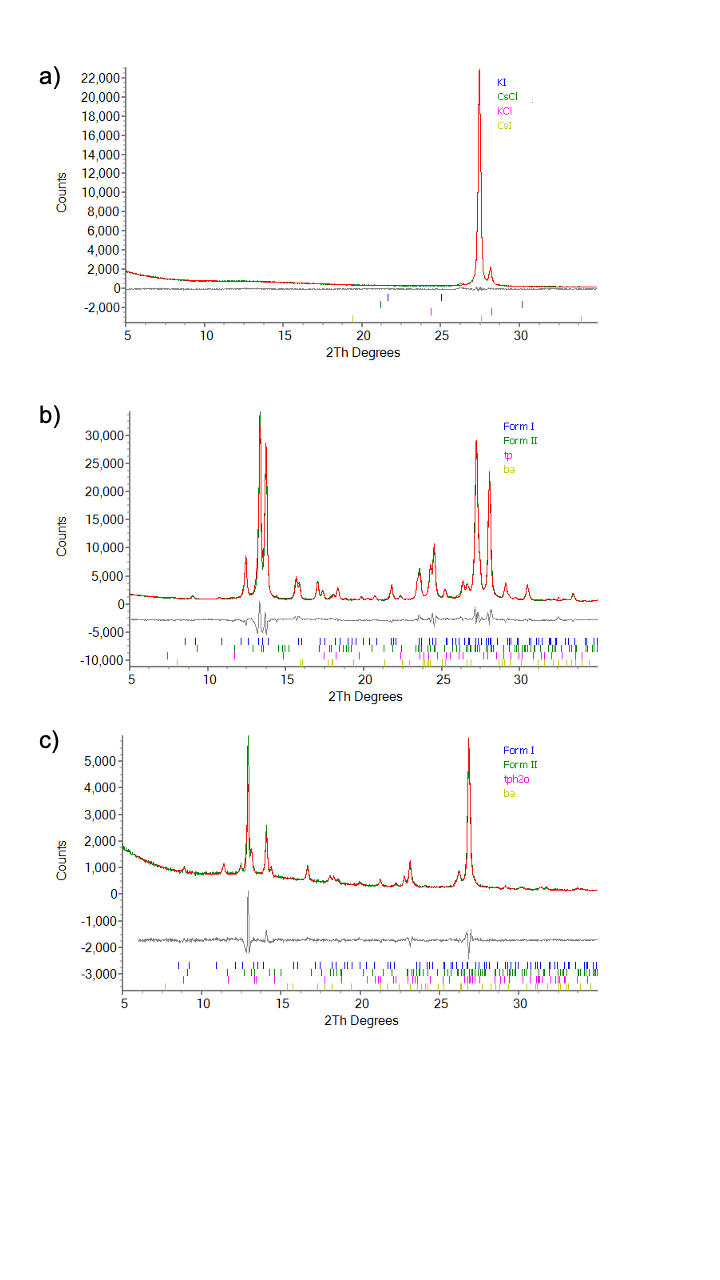


**Supplementary Figure 38|** Representative Rietveld plots for the ex-situ data analysed. Calculated profile (green line) against experimental data (red line) and difference pattern (grey line). Peak marks are shown for all phases as reported in the legends. a) Reaction I, “loosened bottom” powder – see Supplementary Figure 34, section d table. b) Reaction III NG, removed bottom powder – see Supplementary Figure 36, section d table. c) Reaction III LAG: removed bottom powder – see Supplementary Figure 37, section d table.

# **SUPPLEMENTARY NOTE 7| INVESTIGATING PAYLOAD AND BALL SIZE REQUIRED FOR TRIS XRD**

## **Supplementary Note 7.1| Introduction**

We are investigating a new miniaturised design of a 3-component grinding jar with a Perspex segment suitable for in situ synchrotron studies. The grinding jar has a 2.3 mL volume and the thickness of the Perspex segment has been reduced to 0.75 mm (See **Supplementary Figure 5a**).

This study is designed to better understand the optimal grinding parameters with respect to the size of ball and payload of powder best suited for synchrotron-based time resolved in situ (TRIS) X-ray powder diffraction (XRPD).

The study of evaluating the quality of mixing, the homogeneity of the chemical composition product and the kinetics of the ball mill grinding reaction is best done by HPLC analyses, when a covalent chemical reaction is performed. Samples as small as 0.5-1 mg are required for sample preparation, allowing the targeted sampling of many different locations inside the small jar.

The base catalysed disulfide exchange reaction (scheme shown at the top of all figures) is a well understood system and the progress of the mechanochemical reaction can be easily monitored by HPLC because it is a covalent chemical reaction. To make the experiments faster, we pre-milled this reaction for a few minutes at a 2.5 g scale, obtaining 36%M of Form A, with the rest of the contents comprising equimolar amounts of the two homodimers from which this reaction is obtained.

To analyse the distribution of powder over the milling jar after a ball milling reaction, we weighed with a 5-figure balance, the weight of the ball, and the 3 components of the jars before and after completion of the grinding experiments. The distribution of the powder in the 3 components of the jar and the ball was so determined.

The objectives of this exploratory investigation are to:

1. Obtain a suitable rate of mechanochemical reaction

2. Obtain a product with homogenous chemical composition

3. Evaluate the powder that would be available to flow over the transparent Perspex window of the grinding jars.

4. Prevent the powder from caking at the bottom or top cap of the jar.

The supporting data are presented in the following sections:

Supplementary Note 7.3| Correlation between different ball sizes for same loading in milling jar

Supplementary Note 7.4| Correlation between different loading in milling jar for same ball size

Supplementary Note 7.5| Outcome of 30 min NG at 50 Hz: 8.0 mm ss ball - 25 mg – 100 mg payload

Supplementary Note 7.6| Outcome of 30 min NG at 50 Hz: 7.0 mm ss ball – 25 mg – 100 mg payload

Supplementary Note 7.7| Outcome of 30 min NG at 50 Hz: 6.0 mm ss ball – 25 mg – 100 mg payload

Supplementary Note 7.8| Outcome of 30 min NG at 50 Hz: 5.0 mm ss ball – 25 mg – 100 mg payload

Supplementary Note 7.9| Outcome of 30 min NG at 50 Hz: 4.0 mm ss ball – 25 mg – 200 mg payload

## **Supplementary Note 7.2| Overall Conclusions**

The best compromise between homogeneity of the chemical composition, potential powder availability to the transparent Perspex window and the rate of reaction when using the 3-element small jar is the use of a single 8 mm stainless steel ball with a loading of powder between 50 and 75 mg (See **Supplementary Figure 39b**). However, we note if the availability of having free flowing powder along the Perspex segment was not necessary (e.g. for ex situ studies), 25 mg loading would be ideal, as it gave a fast reaction with a very homogenous distribution of the chemical composition.

When using 5 mm or larger milling balls, the chemical composition obtained by HPLC suggests that the powder is being continuously renewed from the walls of the stainless-steel caps as we can achieve quantitative yields (>99%M). The concentration of Form A on the walls of the Perspex segment is not as high (See the red bars in **Supplementary Figures 41-45**). This trend implies that at the early stages of grinding, disulfide powder is pounded by the balls against the soft surfaces of Perspex segment; the totality of this powder may not be scooped away by further impacts of the ball resulting at the end of the grinding experiments in overall lower yields as compared with the powder taken from the walls of stainless steel.

Use of over 100 mg payloads leads to powder caking on the bottom of the jar. Caking, while probably occurring later during ball mill grinding, will trap the starting materials and therefore will result in poor homogeneity of the chemical composition and very poor reaction rates. Moreover, and of particular importance for TRIS XRPD analysis, caking reduces the amount of free-flowing powder that can pass through the beam, thereby reducing the ability to analyse powder in situ.^15^

**Supplementary Note 7.3| Correlation between different ball sizes for same loading in milling jar**

|  |  |
| --- | --- |

**Supplementary Figure 39|** Correlation between different ball sizes (4-8 mm) for same loading (25 to 200 mg) in the small in-house 2.3 mL milling jar. a) left: reaction scheme. Grinding conditions are depicted using pictograms;^14^ right: 3-component grinding jar b) Tabulation and graphical representation of these correlations. In the bar graphs, the total conversion to Form A is shown in blue, and the amount of powder deposited on the Perspex walls is shown in red.

Conclusions from these correlations:

Note: while the trends shown here may be universal, the exact quantities and effects are presumed to be system dependent. Less compressible (more friable) powders will not necessarily follow the same values as a soft and compressible organic powder (e.g. at paracetamol – two polymorphs compress differently).

1. At 25 mg payload, the mechanochemistry reaction is fast achieving a consistent > 98%M Form A when milling with 4 mm to 8 mm balls. Temporary powder deposition on the Perspex segment is low, is it higher when using bigger balls. The use of a 4 mm ball leads to slower reaction rates with payloads between 50 mg and 100 mg.
2. There is a clear trend for experiment with 50 mg payload and above. The use of smaller balls leads to slower rate of reaction than bigger balls.
3. The use of larger balls does not change significantly the temporary powder loading on the Perspex segment. However, temporary loading on the Perspex segment increases from to 25 mg to 50 mg to 75 mg to 100 mg by 1.5-2 times.
4. Using payloads over 100 mg can lead to sereve caking of the powder in the bottom cap; temporary loading of the Perspex segment is small.

The ideal payload sits between 50 mg and 75 mg

## **Supplementary note 7.4| Correlation between different loading in milling jar for same ball size**

|  |  |
| --- | --- |

**Supplementary Figure 40|** Correlation between different loading (25 to 200mg) for same ball sizes (4-8 mm) in the small 2.3 mL in-house milling jar. a) left: reaction scheme. Grinding conditions are depicted using pictograms^14^ and right: design of the 2.3mL milling jar . b) tabulation and graphical representation of these correlations

Conclusions from these correlations:

*Note: while the trends shown here may be universal, the exact quantities and effects are presumed to be system dependent.*

1. 4 mm balls are only effective with small payloads. However, the Perspex segment is poorly covered with powder. The transformation of the chemical reaction is poor with payloads above 50 mg.
2. Caking appears above 100 mg. Although we only demonstrate it here with the 4 mm balls, we have extensive (not reported here) experience that above 100 mg loading caking is always observed even with larger balls.
3. The larger and heavier 7 mm and 8 mm balls are most effective balance between effective grinding and powder coverage of the transparent Perspex jar component, giving comparable results: the kinetics are fast.
4. There is a clear trend for ball sizes from 5-8 mm. Powder covers the Perspex segment better with heavier loading of powder, however the rates of the reactions are slowed down.

A trend can be easily seen with all balls of 5 mm and above: The higher the payload, the slower the reaction rate but with improved powder coating of the Perspex window.

## **Supplementary Note 7.5| Outcome of 30 min NG at 50 Hz: 8.0 mm ss ball- 25 mg – 100 mg payload**

|  |  |
| --- | --- |

**Supplementary Figure 41|** Exploratory work: Outcome of 30 min NG at 50 Hz using a 2.3 mL 3-component grinding jar: 8.0 mm ss ball- 25mg-100 mg payload a) left: reaction scheme. Grinding conditions are depicted using pictograms;^14^ right: locations in grinding jar for taking around 1 mg samples of powder for HPLC analysis: b) tabulation of experimental parameters and pictures of jars opened on completion of milling. Tabulation and graphical representation of c) distribution of powder on top & bottom caps, Perspex clear segment & ss ball; d) homogeneity of the powder: chemical composition by HPLC analysis over many locations as shown in a)

## **Supplementary Note 7.6| Outcome of 30 min NG at 50 Hz: 7.0 mm ss ball- 25 mg – 100 mg payload**

|  |  |
| --- | --- |


**Supplementary Figure 42|** Exploratory work: Outcome of 30 min NG at 50 Hz using a 2.3 mL 3-component grinding jar: 7.0 mm ss ball- 25mg-100 mg payload a) left: reaction scheme. Grinding conditions are depicted using pictograms;^14^ right: locations in grinding jar for taking around 1 mg samples of powder for HPLC analysis: b) tabulation of experimental parameters and pictures of jars opened on completion of milling. Tabulation and graphical representation of c) distribution of powder on top & bottom caps, Perspex clear segment & ss ball; d) homogeneity of the powder: chemical composition by HPLC analysis over many locations as shown in a).

## **Supplementary Note 7.7| Outcome of 30 min NG at 50 Hz: 6.0 mm ss ball- 25 mg – 100 mg payload**

|  |  |
| --- | --- |

**Supplementary Figure 43|** Exploratory work: Outcome of 30 min NG at 50 Hz using a 2.3 mL 3-component grinding jar: 6.0 mm ss ball- 25 mg-100 mg payload a) left: reaction scheme. Grinding conditions are depicted using pictograms;^14^ right: locations in grinding jar for taking around 1 mg samples of powder for HPLC analysis: b) tabulation of experimental parameters and pictures of jars opened on completion of milling. Tabulation and graphical representation of c) distribution of powder on top & bottom caps, Perspex clear segment & ss ball; d) homogeneity of the powder: chemical composition by HPLC analysis over many locations as shown in a).

## **Supplementary Note 7.8| Outcome of 30 min NG at 50 Hz: 5.0 mm ss ball- 25 mg – 100 mg payload**

|  |  |
| --- | --- |

**Supplementary Figure 44|** Exploratory work: Outcome of 30 min NG at 50 Hz using a 2.3 mL 3-component grinding jar: 5.0 mm ss ball- 25mg-100 mg payload a) left: reaction scheme. Grinding conditions are depicted using pictograms;^14^ right: locations in grinding jar for taking around 1 mg samples of powder for HPLC analysis: b) tabulation of experimental parameters and pictures of jars opened on completion of milling. Tabulation and graphical representation of c) distribution of powder on top & bottom caps, Perspex clear segment & ss ball; d) homogeneity of the powder: chemical composition by HPLC analysis over many locations as shown in a).

## **Supplementary note 7.9| Outcome of 30 min NG at 50 Hz: 4.0 mm ss ball- 25 mg – 100 mg payload**

|  |  |
| --- | --- |

**Supplementary Figure 45|** Exploratory work: Outcome of 30 min NG at 50 Hz using a 2.3 mL 3-component grinding jar: 4.0 mm ss ball- 25 mg-200 mg payload a) left: reaction scheme. Grinding conditions are depicted using pictograms;^14^ right: locations in grinding jar for taking around 1 mg samples of powder for HPLC analysis: b & e) tabulation of experimental parameters and pictures of jars opened on completion of milling. Tabulation and graphical representation of c & f) distribution of powder on top & bottom caps, Perspex clear segment & ss ball; d & g) homogeneity of the powder: chemical composition by HPLC analysis over many locations as shown in a).

# **SUPPLEMETNARY NOTE 8| TOPAS CUSTOM MACROS**

macro Peak_split_simple(beam_SD_V, beam_SD)

{

#m_argu beam_SD_V

If_Prm_Eqn_Rpt(beam_SD_V, beam_SD, min = Val-.1; max = Val+.1; del 0.001)

th2_offset = (Rad * ArcSin((CeV(beam_SD_V, beam_SD) * Sin(2 Th))/Rs));

}

macro TCHZ_Split_Peak_Type(u_Left, u_Leftv, v_Leftv, v_Left, w_Left, w_Leftv, z_Left, z_Leftv, x_Left, x_Leftv, y_Left, y_Leftv, u_Right, u_Rightv, v_Right, v_Rightv, w_Right, w_Rightv, z_Right, z_Rightv, x_Right, x_Rightv, y_Right, y_Rightv)

{

#m_argu u_Left

#m_argu v_Left

#m_argu w_Left

#m_argu z_Left

#m_argu x_Left

#m_argu y_Left

#m_argu u_Right

#m_argu v_Right

#m_argu w_Right

#m_argu z_Right

#m_argu x_Right

#m_argu y_Right

If_Prm_Eqn_Rpt(u_Left, u_Leftv, min = Max(-1, Val-.1); max = Min(2, Val+.1); del 1.0e-4)

If_Prm_Eqn_Rpt(v_Left, v_Leftv, min = Max(-1, Val-.1); max = Min(2, Val+.1); del 1.0e-4)

If_Prm_Eqn_Rpt(w_Left, w_Leftv, min = Max(-1, Val-.1); max = Min(2, Val+.1); del 1.0e-4)

If_Prm_Eqn_Rpt(z_Left, z_Leftv, min = Max(-1, Val-.1); max = Min(2, Val+.1); del 1.0e-4)

If_Prm_Eqn_Rpt(x_Left, x_Leftv, min = Max(0.0001, Val-.1); max = Min(2, Val+.1); del 1.0e-4 )

If_Prm_Eqn_Rpt(y_Left, y_Leftv, min = Max(0.0001, Val-.1); max = Min(2, Val+.1); del 1.0e-4 )

If_Prm_Eqn_Rpt(u_Right, u_Rightv, min = Max(-1, Val-.1); max = Min(2, Val+.1); del 1.0e-4)

If_Prm_Eqn_Rpt(v_Right, v_Rightv, min = Max(-1, Val-.1); max = Min(2, Val+.1); del 1.0e-4)

If_Prm_Eqn_Rpt(w_Right, w_Rightv, min = Max(-1, Val-.1); max = Min(2, Val+.1); del 1.0e-4)

If_Prm_Eqn_Rpt(z_Right, z_Rightv, min = Max(-1, Val-.1); max = Min(2, Val+.1); del 1.0e-4)

If_Prm_Eqn_Rpt(x_Right, x_Rightv, min = Max(0.0001, Val-.1); max = Min(2, Val+.1); del 1.0e-4 )

If_Prm_Eqn_Rpt(y_Right, y_Rightv, min = Max(0.0001, Val-.1); max = Min(2, Val+.1); del 1.0e-4 )

local #m_unique tch_p_l_Left = CeV(x_Left, x_Leftv) Tan(Th) + CeV(y_Left, y_Leftv) / Cos(Th);

local #m_unique tch_p_g_Left = Sqrt( Abs( CeV(u_Left, u_Leftv) Tan(Th)^2 + CeV(v_Left, v_Leftv) Tan(Th) + CeV(w_Left, w_Leftv) + CeV(z_Left, z_Leftv) / Cos(Th)^2) );

local #m_unique tch_p_Left =

(

tch_p_g_Left^5 +

2.69269 tch_p_g_Left^4 tch_p_l_Left +

2.42843 tch_p_g_Left^3 tch_p_l_Left^2 +

4.47163 tch_p_g_Left^2 tch_p_l_Left^3 +

0.07842 tch_p_g_Left tch_p_l_Left^4 +

tch_p_l_Left^5

)^0.2;

local #m_unique tch_q_Left = tch_p_l_Left / tch_p_Left;

local #m_unique tch_p_l_Right = CeV(x_Right, x_Rightv) Tan(Th) + CeV(y_Right, y_Rightv) / Cos(Th);

local #m_unique tch_p_g_Right = Sqrt( Abs( CeV(u_Right, u_Rightv) Tan(Th)^2 + CeV(v_Right, v_Rightv) Tan(Th) + CeV(w_Right, w_Rightv) + CeV(z_Right, z_Rightv) / Cos(Th)^2) );

local #m_unique tch_p_Right =

(

tch_p_g_Right^5 +

2.69269 tch_p_g_Right^4 tch_p_l_Right +

2.42843 tch_p_g_Right^3 tch_p_l_Right^2 +

4.47163 tch_p_g_Right^2 tch_p_l_Right^3 +

0.07842 tch_p_g_Right tch_p_l_Right^4 +

tch_p_l_Right^5

)^0.2;

local #m_unique tch_q_Right = tch_p_l_Right / tch_p_Right;

peak_type spv

spv_l1 = 1.36603 tch_q_Left - 0.47719 tch_q_Left^2 + 0.1116 tch_q_Left^3;

spv_h1 = tch_p_Left;

spv_l2 = 1.36603 tch_q_Right - 0.47719 tch_q_Right^2 + 0.1116 tch_q_Right^3;

spv_h2 = tch_p_Right;**SUPPLEMENTARY NOTE 9| GENERAL DISCUSSION**

When considering XRPD, a number of key features must be extractable for reliable analysis. First, accurate positions of the Bragg reflections are necessary to analyse the crystallographic unit cells under investigation. Second, accurate intensities of the reflections provide essential information regarding the atomic structure of the material, and correct phase quantification. Finally, the widths of Bragg reflections depend on the crystallinity of the material; accurate determination of these widths is therefore essential for exploring the microstructure, i.e. the crystallite size and the microstrain. Other features, such as accurate scattering background, are also significant, and can provide crucial insight into the presence of non-crystalline phases.

Typically, the quality of XRPD data derived from TRIS analysis has been suboptimal, leading to significant uncertainties regarding phase identification, crystal structure, particle size, and microstructure. Hence, robust analysis of mechanochemical transformations has been limited to *ex situ* analysis. In the present paper we have made extensive efforts to improve the data collection and processing strategies. Our thin-walled milling jars (0.5-0.7 mm) greatly reduce the background scattering, and hence enhance the scattering signal of the material within the wall. This has allowed us to reduce the quantity of material required for in situ investigation (formerly *ca.* 200-1000 mg) to only 10-60 mg. Simultaneously, the enhanced scattering from the sample increases the resolution of the diffraction profile, leading to improved confidence in the position and shape of diffraction peaks. Associated with the reduced wall thickness, our XRD data was collected at significantly lower energies than usually employed for TRIS measurements. By diffracting at 17 keV (instead of > 40 keV used at other synchrotron sources) our Bragg reflections were highly resolved, thereby avoiding any undesirable overlap of reflections, and thus providing reliable peak shapes. Finally, our data collection strategy allowed for careful alignment of the X-ray beam, thereby reducing artificial splitting of the Bragg reflections from non-ideal sample geometry. Together, these developments allowed us to construct a robust model for the structure of scattering from our set-up, and hence to extract robust and reliable data for full profile analysis. Thinner walls can of course enhance thermal conductivity. However, thermal measurements in jars with 2.5 mm walls suggested global temperature rises of only 5-10 ^o^C over the course of a ball milling reaction.^16^ Thus, any change in thermal conductivity will have negligible effects on the transformation.

The loss of free flowing powder during ball milling is a critical issue for TRIS-XRPD analysis. When powder cakes, clumps, or is otherwise stuck to an internal surface of the jar, it becomes ‘invisible’ to the X-ray beam.^15^ Correspondingly, accurate analysis of reaction profiles requires such effects to be minimised. This is particularly challenging when liquid assisted grinding reactions of highly compressible materials (e.g. organic solids) is being considered. Although we cannot claim to have solved this problem in the present work, Supplementary Note 7 outlines a significant advancement towards reducing this issue. We have found that free flowing powder can be maximised by carefully controlling the fill volume. We strongly encourage all researchers involved in TRIS XRPD for ball milling reactions to conduct such preliminary work to ensure reliability of their data sets.

**SUPPLEMENTARY NOTE 10| NOMENCLATURE AND ABBREVIATIONS USED**

| [ ] | Symbol representing CAS number |
| --- | --- |
| LAG | Ball mill liquid assisted grinding. The term “LAG” is equivalent and assumes we are discussing ball mill LAG |
| NG | Ball mill neat grinding. The term “NG” is equivalent and assumes we are discussing ball mill neat grinding |
| (2NO_2_PhS)_2_ | bis(2-nitrophenyl) disulfide homodimer [1155-00-6]. |
| (4ClPhS)_2_ | bis(4-chlorophenyl) disulfide homodimer [1142-19-4]. |
| Form A | (2NO_2_PhSSPh4Cl) polymorph obtained typically from NG: CSD refcode FUQLIM01. |
| Form B | (2NO_2_PhSSPh4Cl) polymorph obtained typically from LAG (50mL MeCN): CSD refcode FUQLIM. |
| dbu | 1,8-Diazabicyclo[5.4.0]undec-7-ene (base catalyst) [6674-22-2] |
| tp | Theophylline anhydrous [58-55-9] |
| ba | Benzamide [55-21-0] |
| Form I | 1:1 tp:ba Form I polymorph typically obtained by NG;  CSD refcode RABXIE02. Metastable bulk; P4_1_, Z’=2 |
| Form II | 1:1 tp:ba Form II polymorph typically obtained by LAG  CSD refcode RABXIE01. Stable bulk; P2_1_, Z’=1 |
| KI | Potassium iodide [7681-11-0] |
| CsCl | Cesium chloride [7647-17-8] |
| ZIF-8 | Zeolitic Imidazolate Framework 8  CSD refcode VELVOY |
| ZnO | Zinc oxide [1314-13-2] |
| MeIm | 2-Methyl-imidazole [693-98-1] |
| NH_4_NO_3_ | Ammonium nitrate [6484-52-2] |
| DMF | N,N-Dimethylformamide [68-12-2] |
| PMMA | polymethyl methacrylate. Also known as Perspex |
| PVC  MeCN | Polyvinyl chloride  Acetonitrile |
| TFA | Trifluoroacetic acid |
| FA | Formic acid |
| HPLC  GC | High performance liquid chromatography  Gas chromatography |
| XRPD | X-ray Powder Diffraction |
| ID  OD | Internal diameter  Outside diameter |
| Hz | Hertz (frequency used to swing the grinding jars by the ball mill grinder) |
| h | hours |
| m | minutes |
| s | seconds |
| BAM | Federal Institute for Materials Research and Testing in Berlin |
| QPA  CofA | Quantitative Phase Analysis  Certificate of Analysis |

# **SUPPLEMENTARY REFERENCES**

1. Belenguer, A. M., Lampronti, G. I., Wales, D. J. & Sanders, J. K. M. Direct observation of intermediates in a thermodynamically controlled solid-state dynamic covalent reaction. *J. Am. Chem. Soc.* **136**, 16156–16166 (2014).

2. Gozzo, F. *et al.* Instrumental profile of MYTHEN detector in Debye-Scherrer geometry. *Z. Krist.* **225**, 616–624 (2010).

3. Thompson, P., Cox, D. E. & Hastings, J. B. Rietveld refinement of Debye–Scherrer synchrotron X-ray data from Al2O3. *J. Appl. Crystallogr.* **20**, 79–83 (1987).

4. Coelho, A. A. *TOPAS-Academic Version 6 - Technical Reference*. (2016).

5. Cline, J. P., Black, D., Gil, D., Henins, A. & Windover, D. The Application of the Fundamental Parameters Approach as Implemented in TOPAS to Divergent Beam Powder Diffraction Data. *Mater. Sci. Forum* **651**, 201–219 (2010).

6. Black, D. R. *et al.* Certification of NIST Standard Reference Material 640d.

7. Coelho, A. A. TOPAS and TOPAS-Academic: an optimization program integrating computer algebra and crystallographic objects written in C++. *J. Appl. Crystallogr.* **51**, 210–218 (2018).

8. Payzant, E. A. Other Topics. in *Principles and Applications of Powder Diffraction* (eds. Clearfield, A., Reibenspies, J. H. & Nattamai, B.) 365–380 (John Wiley and Sons, Ltd, 2008).

9. Madsen, I. C. & Scarlett, N. V. Y. Quantitative Phase Analysis. in *Powder Diffraction – Theory and Practice* (eds. Dinnabier, R. E. & Billinge, S. J. L.) (RCS publishing, 2008).

10. Stinton, G. W. & Evans, J. S. O. Parametric Rietveld refinement. *J. Appl. Crystallogr.* **40**, 87–95 (2007).

11. Michalchuk, A. A. L., Tumanov, I. A. & Boldyreva, E. V. Complexities of mechanochemistry: Elucidation of processes occurring in mechanical activators via implementation of a simple organic system. *CrystEngComm* **15**, 6403–6412 (2013).

12. Black, D. R., Windover, D., Henins, A., Filliben, J. & Cline, J. P. Certification of standard reference material 660B. *Powder Diffr.* **26**, 155–158 (2011).

13. Cheary, R. W., Coelho, A. A. & Cline, J. P. Fundamental parameters line profile fitting in laboratory diffractometers. *J. Res. Natl. Inst. Stand. Technol.* **109**, 1 (2004).

14. Michalchuk, A. A. L., Boldyreva, E. V, Belenguer, A. M., Emmerling, F. & Boldyrev, V. V. Tribochemistry, mechanical alloying, mechanochemistry: what is in a name? *Front. Chem.* **9**, 359 (2021).

15. Michalchuk, A. A. L. *et al.* Challenges of Mechanochemistry: Is In Situ Real-Time Quantitative Phase Analysis Always Reliable? A Case Study of Organic Salt Formation. *Adv. Sci.* **4**, 1700132 (2017).

16. Kulla, H. *et al.* Tuning the apparent stability of polymorphic cocrystals through mechanochemistry. *Cryst. Growth Des.* **19**, 7271–7279 (2019).
